# Combined Blockade of Lipid Uptake and Synthesis by CD36 Inhibitor and SCD1 siRNA Is Beneficial for the Treatment of Refractory Prostate Cancer

**DOI:** 10.1002/advs.202412244

**Published:** 2024-12-30

**Authors:** Jiyuan Chen, Xiaoyan Yu, Gang Yang, Xueying Chen, Chunai Gong, Lu Han, Yujie Wang, Rong Wang, Lei Wang, Yongfang Yuan

**Affiliations:** ^1^ Department of Pharmacy Shanghai Ninth People's Hospital Shanghai Jiao Tong University School of Medicine Shanghai 200011 P. R. China; ^2^ Department of Stomatology The Sixth Affiliated Hospital of Sun Yat‐Sen University Guangzhou 510655 P. R. China

**Keywords:** CD36, drug resistance, lipid metabolism, refractory prostate cancer, SCD1

## Abstract

Drug resistance is an important factor for prostate cancer (PCa) to progress into refractory PCa, and abnormal lipid metabolism usually occurs in refractory PCa, which presents great challenges for PCa therapy. Here, a cluster of differentiation 36 (CD36) inhibitor sulfosuccinimidyl oleate sodium (CD36i) and stearoyl‐CoA desaturase 1 (SCD1) siRNA (siSCD1) are selected to inhibit lipid uptake and synthesis in PCa, respectively. To this end, a multiresponsive drug delivery nanosystem, HA@CD36i‐TR@siSCD1 is designed. The hyaluronic acid (HA) gel “shell” of HA‐TR nanosystem can release drugs in response to the acidic tumor microenvironment and hyaluronidase, and the tumor targeting (TR) cationic micellar “core” can release drugs in response to glutathione. This multiresponsive drug release is beneficial for the exogenous inhibition of lipid uptake by CD36i and the endogenous inhibition of lipid synthesis by siSCD1. The established HA‐TR nanosystem has good tumor targeting ability and tumor penetration ability, and that HA@CD36i‐TR@siSCD1 has good synergistic effects, which can significantly restrain the growth, invasion, and metastasis of PCa. Moreover, under high‐fat conditions, the tumors are more sensitive to HA@CD36i‐TR@siSCD1 treatment, almost no accumulation of lipid droplets is observed in HA@CD36i‐TR@siSCD1‐treated tumors, with enhanced antitumor immunity. Hence, this study provides a new treatment option for refractory PCa patients, especially those with a high‐fat diet.

## Graphical abstract

The established HA@CD36i‐TR@siSCD1 nanosystem actively targets prostate cancer (PCa) and is multiresponsive to release cluster of differentiation 36 (CD36) inhibitor in tumor microenvironment and release stearoyl‐CoA desaturase 1 (SCD1) siRNA in tumor cell, respectively, to regulate lipid uptake and synthesis of PCa. Additionally, HA@CD36i‐TR@siSCD1 can inhibit regulatory T cells, thereby enhancing anti‐tumor immunity, and synergically inhibit tumor growth and metastasis.

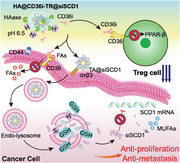

## 1 Introduction

Prostate cancer (PCa) is the second most common cancer and the fifth most mortal cancer among men worldwide.^[^ ^1^ ^]^ Despite the extremely high 5‐year survival rate for primary prostate cancer (97%), second only to thyroid cancer (99%), castration‐resistant prostate cancer (CRPC) is fatal, aggressive, and highly metastatic, with a 5‐year survival rate of less than 30%.^[^ ^2^, ^3^, ^4^ ^]^ Enzalutamide (Enz), one of the frontline treatments for CRPC and metastatic CRPC (mCRPC), can prolong the survival of CRPC and mCRPC patients.^[^ ^5^ ^]^ However, the development of enzalutamide resistance has led to the progression of PCa into refractory PCa, with few clinical options and a poor prognosis.^[^ ^6^ ^]^

Aberrant lipid metabolism has been widely observed in PCa, including abnormalities in lipid synthesis, oxidation, storage and transport.^[^ ^7^ ^]^ These abnormalities can affect biological functions such as proliferation, metastasis, and drug resistance in PCa, thereby exacerbating the progression and spread of PCa and eventually progressing to refractory PCa.^[^ ^8^ ^]^ The inhibition of lipid synthesis via the inhibition of fatty acid synthase (FASN) can induce DNA damage in PCa and shows a good synergism with PARP inhibitors.^[^ ^9^ ^]^ Sterol regulatory element‐binding protein (SREBP) is the upstream regulator of FASN, and we previously reported that SREBP1 siRNA can significantly inhibit the expression of SREBP1 and its downstream factor stearoyl‐CoA desaturase 1 (SCD1) and improve the treatment and prognosis of mice with bone metastatic CRPC in combination with docetaxel.^[^ ^10^ ^]^ The downstream factor SCD1 is the main rate‐limiting enzyme for the synthesis of monounsaturated fatty acids (MUFAs), including oleic acid (OA). SCD1 can reduce fatty acid oxidation and increase lipid synthesis and ferroptosis resistance, thereby promoting cancer cell growth.^[^ ^11^, ^12^, ^13^ ^]^ Moreover, SCD1 inhibitors can delay Enz resistance and PCa progression.^[^ ^14^ ^]^ In addition, cluster of differentiation 36 (CD36) is reportedly overexpressed in tumors and is associated with malignant progression and metastasis. CD36 is a cell membrane receptor that mediates the uptake of fatty acids (FAs) by cells. Owing to the high energy requirements of tumor cells, CD36 helps tumor cells take up large amounts of lipids to maintain their growth and metastasis.^[^ ^15^, ^16^ ^]^ In addition, CD36 is highly expressed in a variety of immunosuppressive cells, such as regulatory T (Treg) cells and tumor‐associated macrophages (TAMs), thereby inhibiting antitumor immunity and forming immune “cold” tumors.^[^ ^16^, ^17^ ^]^ Wang et al. reported that CD36 can promote the proliferation of Treg cells by mediating PPAR‐β, thus intensifying the immunosuppressive microenvironment of tumors.^[^ ^17^ ^]^ In contrast, CD36 overexpression in tumors leads to dysfunction of dendritic cells (DCs) and CD8^+^ T cells, which further intensifies the formation of an immunosuppressive microenvironment.^[^ ^18^, ^19^ ^]^ Additionally, after the combination of surgical castration and Enz treatment, *Cd36* was one of the genes whose expression was upregulated the most in *Pten^L/L^ Smad4^L/ L^* mice.^[^ ^20^ ^]^ CD36 inhibition combined with Enz can promote tumor apoptosis and inhibit tumor proliferation in high‐fat diet (HFD)‐fed PCa‐bearing mice.^[^ ^21^ ^]^ Therefore, the combined blockade of CD36 and SCD1 is expected to inhibit the lipid uptake and lipid synthesis in tumor cells, eliminate the important energy source of PCa, and synergistically inhibit the growth and metastasis of Enz‐resistant refractory PCa, providing a new therapeutic option for the treatment of refractory PCa.

**Scheme 1:**
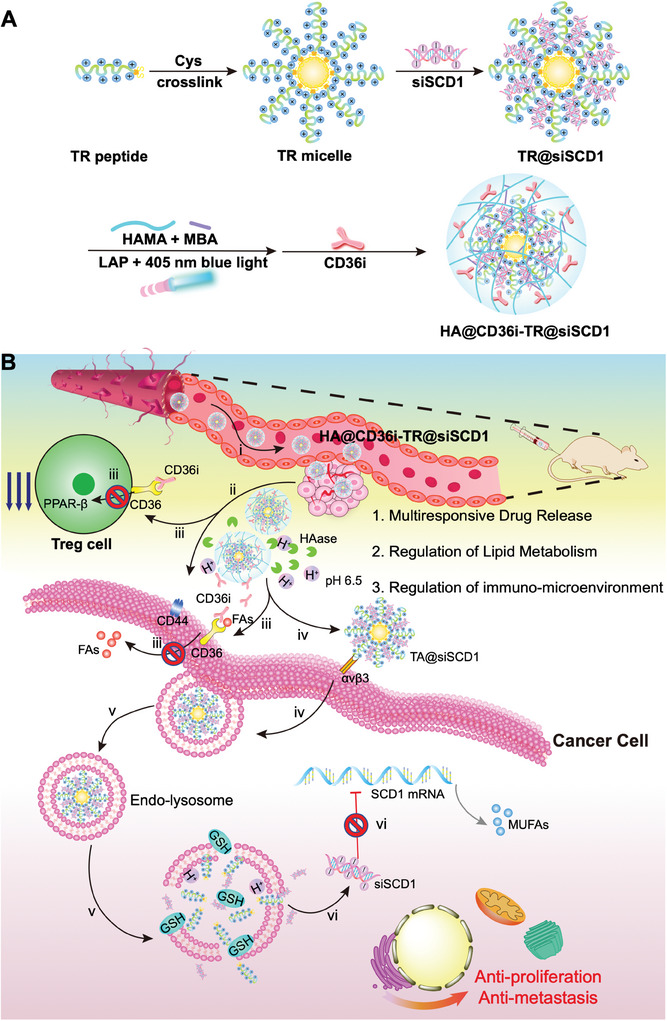
Schematic illustration of the A) establishment and B) mechanism of HA@CD36i‐TR@siSCD1 nanosystem.

Herein, we developed a novel multiresponsive drug‐releasing “core‐shell” nanosystem (HA@CD36i‐TR@siSCD1) for the codelivery of a CD36 inhibitor (sulfosuccinimidyl oleate sodium, CD36i) and SCD1 siRNA (siSCD1) (**Scheme** **1**A). The “core” of this nanocomposite is a self‐assembled tumor targeting (TR) peptide micelles loaded with siSCD1 (TR@siSCD1). The TR peptide (LA‐ rkkrrqrrrHlKYDGR, where LA is lipoic acid, and the lowercase letter indicates that the amino acid is a D‐type amino acid) is designed for tumor‐targeted delivery of siSCD1 and consists of the short RGD peptide and the cationic cell‐penetrating peptide Tat9, which is beneficial for tumor targeting and penetration as well as for gene compression and transfection.^[^ ^22^, ^23^, ^24^ ^]^ Moreover, the Tat9 peptide is composed of D‐type amino acids, which may reduce the potential toxicity of cationic polypeptides.^[^ ^25^ ^]^ In addition, the TR peptide is modified by lipoic acid at the N‐terminus, which can be crosslinked with cysteine to form disulfide bonds and is sensitive to high intracellular concentrations of glutathione in PCa.^[^ ^26^ ^]^ The “shell” is a self‐assembled (under 405 nm blue light) hyaluronic acid (HA) gel with pH‐sensitive acrylamide bonds for the encapsulation of CD36i, which can target the cluster of differentiation 44 (CD44) receptor in the tumor microenvironment (TME) and release drugs in response to hyaluronidase (HAase) and acidic pH in the TME.^[^ ^27^, ^28^ ^]^

As displayed in Scheme 1B, after the administration of HA@CD36i‐TR@siSCD1 via tail vein, i) HA@CD36i‐TR@siSCD1 actively targets PCa in response to high expression of CD44 in PCa cells and accumulates at the tumor site; ii) the HA@CD36i gel shell releases CD36i in response to the acidic TME (pH 6.5) and high concentration of HAase; iii) CD36i inhibits Treg cell function and lipid uptake in tumor cells, respectively; iv) TR@siSCD1 micelles target integrin receptor αvβ3 on tumor cells and then are ingested by PCa cells; v) TR@siSCD1 micelles escape from the endolysosomes through the proton sponge effect and release siSCD1 into the cytoplasm in response to high concentrations of glutathione (GSH) in tumor cells; and vi) siSCD1 targets SCD1 mRNA and inhibits its translation and downstream regulation, thereby inhibiting the proliferation and metastasis of PCa cells in combination with CD36i. To verify the above hypothesis, we first constructed and evaluated the HA@CD36i‐TR@siSCD1 nanosystem, and then investigated the in vitro and in vivo transport mechanisms of HA@CD36i‐TR@siSCD1. In addition, the Enz‐resistant cell Lines C4‐2B_Enz_ and RM‐1_Enz_ were selected for the following study, and 2D/3D cell models with or without high concentrations of OA as well as low‐fat diet (LFD)‐fed and high‐fat diet (HFD)‐fed mouse models were established to investigate the efficacy and safety of HA@CD36i‐TR@siSCD1 in vitro and in vivo, with the goal of providing a new treatment option for patients with refractory PCa, especially obese patients on a HFD.

## 2 Results

### 2.1 The HA‐TR Nanosystem is Multiresponsive for Drug Release and has Good Gene Carrying Ability, Transfection ability, Safety, and Stability

**Figure 1:**
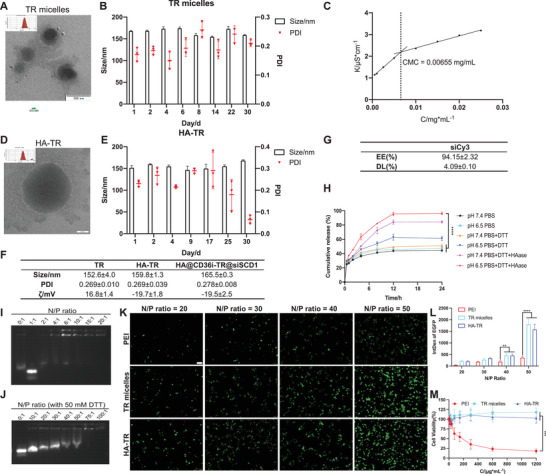
Characterization of TR micelles and HA‐TR nanoparticles. A) Transmission electron microscope (TEM) image of TR micelles (bar = 200 nm); B) Stability of TR micelles in PBS buffer (pH 7.4) at 4 °C for 30 days (n = 3, mean ± SD); C) CMC of TR micelles; D) TEM image of HA‐TR nanoparticles (bar = 50 nm); E) Stability of HA‐TR in PBS buffer (pH 7.4) at 4 °C for 30 days (n = 3, mean ± SD); F) The particle sizes and zeta potentials of TR micelles, HA‐TR and HA@CD36i‐TR@siSCD1 (n = 3, mean ± SD); G) EE% and DL% for siCy3 in HA@‐TR@siCy3 (n = 3, mean ± SD); H) Cumulative release curve of siCy3 (HA‐TR@siCy3) in PBS buffers (pH = 6.5 or 7.4) with or without DTT/HAase (n = 3, mean ± SD, two‐way ANOVA, ^****^ *p* < 0.0001); I) Agarose gel electrophoresis with different N/P ratios TR@pEGFP; J) Agarose gel electrophoresis with different N/P ratios TR@pEGFP with 50 mM DTT; K,L) HEK‐293T cells were co‐incubated with different N/P ratios TR@pEGFP or HA‐TR@pEGFP for 24 h and witnessed under a fluorescence microscope, PEI@pEGFP was used as a positive control (bars = 100 µm, n = 3, mean ± SD, two‐way ANOVA, ^**^ *p* < 0.01); M) Vector toxicity investigation, concentration range: 0–1200 µg mL^−1^ (n = 3, mean ± SD, one‐way ANOVA, ^***^ *p* < 0.001).

The TR peptide was synthesized and purified according to the synthesis route shown in Figure S1 (Supporting Information), with a purity > 95% and a molecular weight of 2397.95 (Figures S2 and S3, Supporting Information). In addition, the sequence of the peptide rkkrrqrrrHlKYDGR was identified via mass spectrometry (Figure S4, Supporting Information). Next, TR micelles were self‐assembled in PBS buffer (pH 7.4) at room temperature. As shown in **Figure** **1**A, the TR micelles were spheroid nanoparticles with a particle size of ≈150 nm. The TR micelles were stable at 4 °C for 30 days (Figure 1B), and the critical micelle concentration (CMC) of the TR micelles was 0.0065 mg mL^−1^ (Figure 1C). Subsequently, hyaluronate methacrylate (HAMA), lithium phenyl‐(2,4,6‐trimethylbenzoyl)‐phosphinate (LAP) and N,N’‐methylenebis(acrylamide) (MBA) were dissolved in TR micelle solution, and the HA‐TR nanoparticles were formed under continuous stirring and 405 nm blue light. The HA‐TR nanoparticles were spheroid nanoparticles with a particle size of ≈150 nm and were stable at 4 °C for 30 days (Figure 1D,E). After the TR micelle core was encapsulated with the HA gel shell, the particle size increased slightly (from 152.6 ± 4.0 nm to 159.8 ± 1.3 nm) and the zeta potential changed from positive (16.8 ± 1.4 mV) to negative (−19.7 ± 1.8 mV), the particle size and zeta potential increased slightly after the loading of CD36i and siSCD11, and the polydispersity index (PDI) of all the nanosystems was less than 0.3, indicating good dispersion (Figure 1F). The drug encapsulation efficiency (EE) and drug loading (DL) rates of the model drug Cy3‐labeled scramble siRNA (siCy3) were 94.15% ± 2.32% and 4.09% ± 0.10%, respectively (Figure 1G). Moreover, to investigate the in vitro drug release behavior of the HA‐TR nanosystem, siCy3 was used as a model drug encapsulated in HA‐TR (HA‐TR@siCy3). Dialysis bags containing HA‐TR@siCy3 were placed in different release systems to observe the cumulative release rate of siCy3 within 24 h. As shown in Figure 1H, since the HA gel shell was sensitive to HAase and the disulfide bond in the TR micelle core was sensitive to the reducing agent dithiothreitol (DTT), the 24 h cumulative release rate of the release system containing DTT and/or HAase was higher than that of the system without DTT and HAase. Notably, in the pH 7.4 + DTT system, the cumulative release rate of siCy3 did not increase significantly, mainly because in the absence of HAase, DTT had difficulty penetrating into the HA‐TR@siCy3 kernel to promote TR micelle decrosslinking. In addition, the cumulative release rate of HA‐TRs at pH 6.5 was greater than that at pH 7.4, indicating a certain acid sensitivity. Therefore, the HA‐TR nanosystem can respond to acidic microenvironments as well as high levels of HAase and reducing agents (e.g., GSH) to release drugs. In addition, the gene compression and transfection ability of TR micelles were evaluated (Figure 1I–L). When the N/P ratio was ≥ 10, the enhanced green fluorescent protein (pEGFP) gene model drug plasmid was completely compressed by the TR micelles (Figure 1I). In the presence of 50 mM DTT, the TR micelles could not compress pEGFP completely until the N/P ratio reached 100, indicating reduced sensitivity (Figure 1J). Moreover, the transfection ability of TR micelles and HA‐TRs was significantly greater than that of the cationic control material polyethyleneimine (PEI), increasing with increasing N/P ratio, and an N/P ratio of 50 was selected as the subsequent drug loading ratio (Figure 1K,L). TR micelles and HA‐TR nanoparticles showed no significant toxicity to HEK‐293T cells in the 0–1200 µg mL^−1^ concentration range, whereas PEI concentrations ≥ 150 µg mL^−1^ resulted in significant cytotoxicity (Figure 1M). In addition, HA‐TR nanoparticles also had no significant toxic effects on PC‐3_Enz_, RM‐1_Enz_, or C4‐2B_Enz_ cells, whereas the Enz‐resistant cell lines PC‐3_Enz_ and RM‐1_Enz_ were constructed as previously reported ^[^ ^29^ ^]^ (Figures S5 and S6, Supporting Information).

### 2.2 The HA‐TR Nanosystem can Escape Endolysosome Phagocytosis, and has Good Cellular Uptake and Tumoroid Penetration Ability

**Figure 2:**
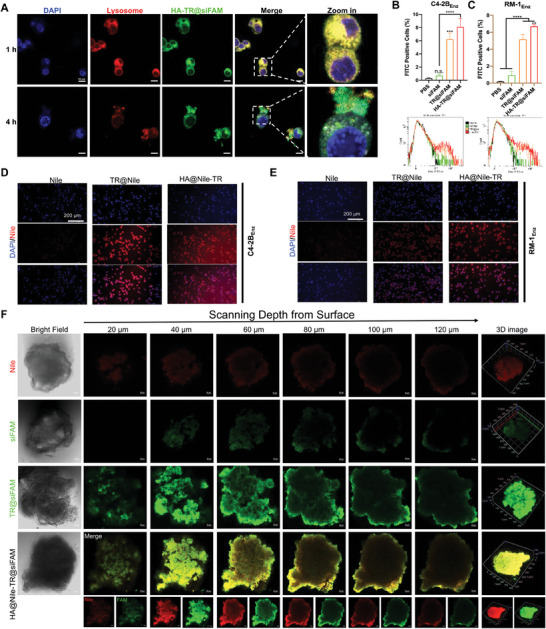
Intracellular uptake and transport of HA‐TR, Nile, and siFAM were used as model drugs. A) Lysosomal escape experiment of HA‐TR@siFAM (bars = 10 µm); B,C) Cellular uptake of HA‐TR@siFAM by C4‐2B_Enz_ or RM‐1_Enz_ cells, analyzed by a flow cytometry (n = 3, mean ± SD, one‐way ANOVA, n.s.: no significance, ^**^ *p* < 0.01, ^***^ *p* < 0.001, ^****^ *p* < 0.0001); D,E) Cellular uptake of HA‐TR@Nile by C4‐2B_Enz_ or RM‐1_Enz_ cells, analyzed by a fluorescence microscope (bars = 200 µm); F) Study on tumoroid penetration ability of HA@Nile‐TR@siFAM in C4‐2B_Enz_ tumoroids (bars = 50 µm).

Free nucleic acid drugs are unstable in the intracellular endolysosome system and can be easily degraded and eliminated.^[^ ^30^ ^]^ Cationic materials can break the membrane of endolysosomes through the “proton sponge effect” to release nucleic acid drugs and prevent their degradation, which is called lysosomal escape.^[^ ^31^ ^]^ After coincubation with HA‐TR@siFAM for 1 h, the green fluorescence of siFAM overlapped with the red fluorescence of the lysosomes, indicating that HA‐TR@siFAM was engulfed by the lysosomes. At 4 h, the green fluorescence of siFAM was separated from the red fluorescence of the lysosomes, which means that siFAM was released from the lysosomes, indicating lysosomal escape ability (**Figure** **2**A). Moreover, siFAM and Nile red (Nile) were used as model drugs to investigate the cellular uptake of TR micelles and HA‐TR nanoparticles. Both TR micelles and HA‐TR nanoparticles significantly increased drug uptake by approximately three and fourfold, respectively (Figure 2B–E). In addition, tumoroids of C4‐2B_Enz_ and RM‐1_Enz_ cells were established to evaluate the tumor penetration ability of TR micelles and HA‐TR nanoparticles. As shown in Figure 2F and Figure S7 (Supporting Information), it was difficult for free drugs to enter the depth of the tumoroids, and the fluorescence of free siFAM and Nile was easily quenched. When drugs are encapsulated by TR micelles or HA‐TR nanoparticles, siFAM and Nile can penetrate into the center of tumoroids, resulting in good tumor penetration ability.

### 2.3 HA@CD36i‐TR@siSCD1 can Significantly Inhibit the Proliferation, Invasion, Metastasis, and Lipid Accumulation of Refractory PCa Cells

**Figure 3:**
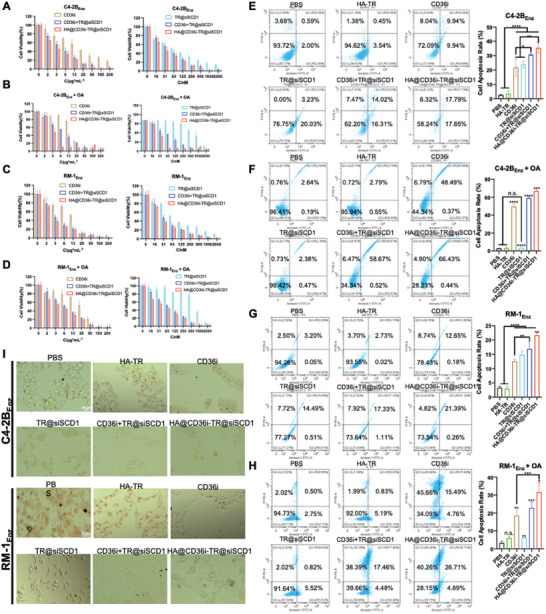
In vitro anti‐proliferation ability of HA@CD36i‐TR@siSCD1. A–D) The anti‐cell proliferation effect of HA@CD36i‐TR@siSCD1 was investigated by a CCK‐8 kit, co‐incubated with (A) C4‐2B_Enz_ cells (B) C4‐2B_Enz_ cells + 500 µM OA (C) RM‐1_Enz_ cells (D) RM‐1_Enz_ cells + 500 µM OA; E–H) The apoptosis effect of HA@CD36i‐TR@siSCD1 was investigated by an Annexin V‐FITC/PI Apoptosis kit, co‐incubated with (E) C4‐2B_Enz_ cells (F) C4‐2B_Enz_ cells + 500 µM OA (G) RM‐1_Enz_ cells (H) RM‐1_Enz_ cells + 500 µM OA (n = 3, mean ± SD, one‐way ANOVA, n.s.: no significance, ^*^ *p* < 0.05, ^**^ *p* < 0.01, ^***^ *p* < 0.001, ^****^ *p* < 0.0001); I) C4‐2B_Enz_ or RM‐1_Enz_ cells were co‐incubated with 500 µM OA and treated with HA@CD36i‐TR@siSCD1 and other controls for 24 h and dyed with oil red O (bars = 20 µm).

Compared with sensitive cell lines, C4‐2B _Enz_ (38.27% ± 0.47%) and RM‐1_Enz_ (44.97% ± 0.64%) cell lines highly expressed CD36 (Figure S8, Supporting Information). Moreover, SCD1 is overexpressed in advanced prostate cancer. The inhibition of SCD1 or CD36 alone can hinder the growth and metastasis of PCa cells,^[^ ^14^, ^21^ ^]^ and its synergistic anti‐proliferative effect was further investigated in this study. As shown in **Figure** **3**A, for C4‐2B_Enz_ cells, CD36i and TR@siSCD1 alone had high cytotoxicity, and the combination of CD36i and TR@siSCD1 had stronger cytotoxicity, with IC_50_ values that were 4.30‐fold and 1.58‐fold lower than those of monotherapy with CD36i or TR@siSCD1, respectively. For the HA@CD36i‐TR@siSCD1 codelivery system, the cell inhibition rate was further decreased, and the IC_50_ value was 1.46‐fold lower than that of the CD36i+TR@siSCD1 system, where the IC_50_ values of CD36i and siSCD1 were 2.915 µg mL^−1^ and 29.15 nM, respectively (Table S1, Supporting Information). For RM‐1_Enz_ cells, the IC_50_ values of the CD36i+TR@siSCD1 group were 2.86‐fold and 2.62‐fold lower than those of the CD36i and TR@siSCD1 monotherapy groups, respectively, and the IC_50_ value of the HA@CD36i‐TR@siSCD1 group was 1.29‐fold lower than that of the CD36i+TR@siSCD1 group (Figure 3C; Table S3, Supporting Information). Furthermore, C4‐2B_Enz_ and RM‐1_Enz_ were coincubated with oleic acid (OA) for 24 h. As shown in Figure 3B,D, the cell inhibition rate of the siSCD1 monotherapy group (TR@siSCD1) was significantly decreased due to exogenous supplementation with OA, the downstream product of SCD1, and the IC_50_ values of C4‐2B_Enz_ and RM‐1_Enz_ cells were 855.2 and 746.6 nM, respectively. The IC_50_ value increased by 12.68‐fold and 8.18‐fold compared with that in the absence of OA. However, CD36i can inhibit CD36 and thus inhibit the cellular uptake of exogenous lipids. Hence, the IC_50_ value of the CD36i group did not change significantly. Nevertheless, C4‐2B_Enz_ and RM‐1_Enz_ cells were highly sensitive to the combination of CD36i and siSCD1 even when supplemented with exogenous. For C4‐2B_Enz_ cells, the IC_50_ values of the CD36i+TR@siSCD1 group were 2.40‐fold and 11.58‐fold lower than those of the CD36i and TR@siSCD1 groups, respectively, and the IC_50_ value of the HA@CD36i‐TR@siSCD1 group was 2.43‐fold lower than that of the CD36i+TR@siSCD1 group. For RM‐1_Enz_ cells, the IC_50_ values of the CD36i+TR@siSCD1 group were 3.13‐fold and 11.00‐fold lower than those of the CD36i and TR@siSCD1 groups, respectively, and the IC_50_ value of the HA@CD36i‐TR@siSCD1 group was 1.66‐fold lower than that of the CD36i+TR@siSCD1 group (Tables S2 and S4, Supporting Information). Additionally, we applied CompuSyn software (http://www.combosyn.com/feature.html) to calculate the combination index (CI) of CD36i and siSCD1 in the CD36i+TR@siSCD1 and HA@CD36i‐TR@siSCD1 groups in all cytotoxicity experiments (Figure S9; Table S5, Supporting Information). In all the tests, the CD36i+TR@siSCD1 and HA@CD36i‐TR@siSCD1 groups exhibited synergistic effects, and the synergistic effect in the HA@CD36i‐TR@siSCD1 group was greater than that in the CD36i+TR@siSCD1 group. In the absence of OA, the CD36i+TR@siSCD1 and HA@CD36i‐TR@siSCD1 groups presented moderate synergism; whereas in the presence of OA, the CD36i+TR@siSCD1 and HA@CD36i‐TR@siSCD1 groups mainly presented synergism, and for C4‐2B_Enz_ cells, the HA@CD36i‐TR@siSCD1 group presented strong synergism, with a CI value of 0.19.

The results of the tumor killing experiment were consistent with those of cytotoxicity experiment. In the absence of OA, the tumoroids disintegrated to varying degrees in both the monotherapy groups and the combination groups, and the tumoroids in the HA@CD36i‐TR@siSCD1 group were completely disintegrated (Figure S10, Supporting Information). In the presence of OA, the tumoroids grew very rapidly, with a diameter of ≈3 mm within one week. For such large tumoroids, no tumoricidal effect was observed in the TR@siSCD1 group, whereas the CD36i and the combination groups presented different degrees of killing effect, with the strongest killing effect observed in the HA@CD36i‐TR@siSCD1 group (Figure S11, Supporting Information).

The results of the apoptosis experiments were consistent with the results of the cytotoxicity experiments (Figure 3E–H; Figure S12, Supporting Information). The apoptotic effects in the combination groups (CD36i+TR@siSCD1 and HA@CD36i‐TR@siSCD1) were greater than those in the monotherapy groups, and the apoptotic effect in the HA@CD36i‐TR@siSCD1 group was the strongest. In the presence of OA, the apoptotic effect in the TR@siSCD1 group was significantly weakened (*p* < 0.001), but C4‐2_Enz_ and RM‐1_Enz_ cells were significantly more sensitive to CD36i and the combined treatment in the presence of OA, and the apoptosis rates were significantly increased (*p* < 0.01). This may be the case when exogenous lipids become the main source of energy and when the blockade of CD36i for lipid uptake by tumor cells becomes a stumbling block for their sustained growth.

This finding was further verified by the oil red O staining experiment. In the absence of OA, only the PBS and HA‐TR groups presented a small amount of lipid accumulation, whereas the other treatment groups presented almost no lipid accumulation and showed a significant reduction in cell proliferation. The HA@CD36i‐TR@siSCD1 group even exhibited extensive cell apoptosis and necrosis (Figure S13, Supporting Information). In the presence of OA, there were many lipid droplets in the PBS and HA‐TR groups, and the number of lipid droplets in the other groups was reduced to varying degrees, and cell proliferation was also reduced correspondingly (Figure 3I).

**Figure 4:**
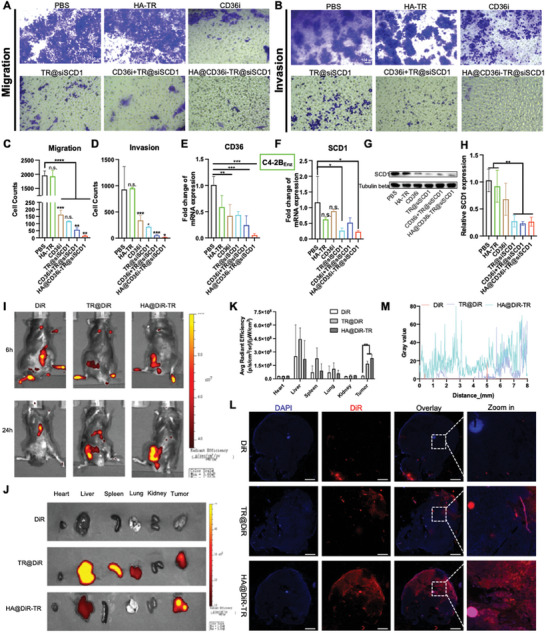
In vitro anti‐invasion and metastasis as well as gene inhibition/knockout investigations, and in vivo distribution study. A) Anti‐metastasis test and C) statistical analysis (bars = 100 µm, n = 3, mean ± SD, multiple *t* test, n.s.: no significance, ^**^ *p* < 0.01, ^***^ *p* < 0.001, ^****^ *p* < 0.0001); B) Anti‐invasion test and D) statistical analysis (bars = 100 µm, n = 3, mean ± SD, multiple *t* test, n.s.: no significance, ^*^ *p* < 0.015, ^***^ *p* < 0.001); E,F) RT‐qPCR analysis of (E) CD36 and (F) SCD1 mRNA levels in C4‐2B_Enz_ cells (n = 3, mean ± SD, one‐way ANOVA, n.s.: no significance, ^*^ *p* < 0.05, ^**^ *p* < 0.01, ^***^ *p* < 0.001); G,H) Western blotting analysis of SCD1 protein expression in C4‐2B_Enz_ cells (n = 3, mean ± SD, one‐way ANOVA, ^**^ *p* < 0.01); I) In vivo fluorescence imaging of RM‐1_Enz_ tumor‐bearing C57BL/6J mice after intravenous injection of DiR, TR@DiR, and HA@DiR‐TR; J) *Ex vivo* imaging of tumors and major organs and K) statistical analysis (n = 3, mean ± SD, multiple *t* test, ^**^ *p* < 0.01); L) Tumor penetration study (bars = 1 mm) and M) statistical analysis.

Additionally, HA@CD36i‐TR@siSCD1 had strong antimetastatic and anti‐invasive effects in vitro, which were better than those of the monotherapy groups (*p* < 0.01) and the CD36i+TR@siSCD1 group (*p* < 0.05) (**Figure** **4**A–D). Moreover, both CD36i and siSCD1 significantly inhibited their respective targets. In the CD36i‐treated groups, the CD36 mRNA levels were significantly decreased (*p* < 0.001); correspondingly, in the siSCD1‐treated groups, the SCD1 mRNA levels were significantly decreased (*p* < 0.05) (Figure 4E,F; Figure S14, Supporting Information). In addition, the expression of SCD1 protein in the HA@CD36i‐TR@siSCD1 group was significantly decreased (Figure 4G,H). These results indicated that the antiproliferative, antimetastatic, anti‐invasive effects and anti‐lipid accumulation effects of HA@CD36i‐TR@siSCD1 on resistant cells or tumoroids were achieved through the regulation of CD36 and SCD1.

### 2.4 The HA‐TR Nanosystem Showed Good Tumor Targeting, Tumor Penetration, and Long Circulation Effects

In vitro cell experiments verified that TR micelles and the HA‐TR nanosystem had good PCa cell‐targeting ability. To further verify their in vivo targeting ability, we established a subcutaneous RM‐1_Enz_‐bearing C57BL/6J mouse model for biodistribution studies. As shown in Figure 4I, after 6 h of tail vein administration, free DiR was distributed mainly in the liver and spleen, whereas TR@DiR and HA‐TR@DiR were distributed mainly at the tumor sites. In addition, there was partial accumulation of fluorescence in the limbs of the three groups of mice, which may be due to partial drug extravasation during the injection of the tail vein, resulting in accumulation of fluorescence on the paws of the hind limbs. After 24 h, free DiR was distributed mainly in the areas of the liver and spleen, while TR@DiR and HA‐TR@DIR continued to accumulate at the tumor site, and the fluorescence intensity increased significantly, suggesting that both TR@DiR and HA‐TR@DiR had long circulation effects and could continue to accumulate at the tumor sites (Figure 4J). In addition, the in vitro imaging of organs revealed that the DiR group mainly accumulated in the liver, spleen and lung, but the overall fluorescence was lower than that of the TR@DiR and HA‐TR@DiR groups, which may be due to the rapid metabolism of free DiR by the liver. The distribution of the TR@DiR and HA‐TR@DiR groups at the tumor sites was significantly greater than that of the free DiR group (*p* < 0.01). In addition, the accumulation of TR@DiR in the liver and spleen was greater than that of HA‐TR@DiR, possibly because the TR micelles had a positive potential and were more likely to be phagocytized and ingested by the mononuclear macrophage system of the liver and spleen, whereas the HA‐TR nanosystem had negative potential and could reduce the phagocytosis and ingestion effects of the mononuclear macrophage system (Figure 4K). Further full‐section fluorescence scanning of the tumor tissues revealed that the DiR group only presented a small amount of fluorescence distributed in the tumor margin, whereas the TR@DiR and HA‐TR@DiR groups presented fluorescence distributed in the tumor interior, and the HA‐TR@DiR group penetrated deep into the tumor, indicating that both the TR micelles and the HA‐TR nanosystem had good tumor penetration ability (Figure 4L,M).

### 2.5 HA@CD36i‐TR@siSCD1 Inhibited Tumor Progression and Metastasis and Prolonged the Survival of Tumor‐Bearing Mice

**Figure 5:**
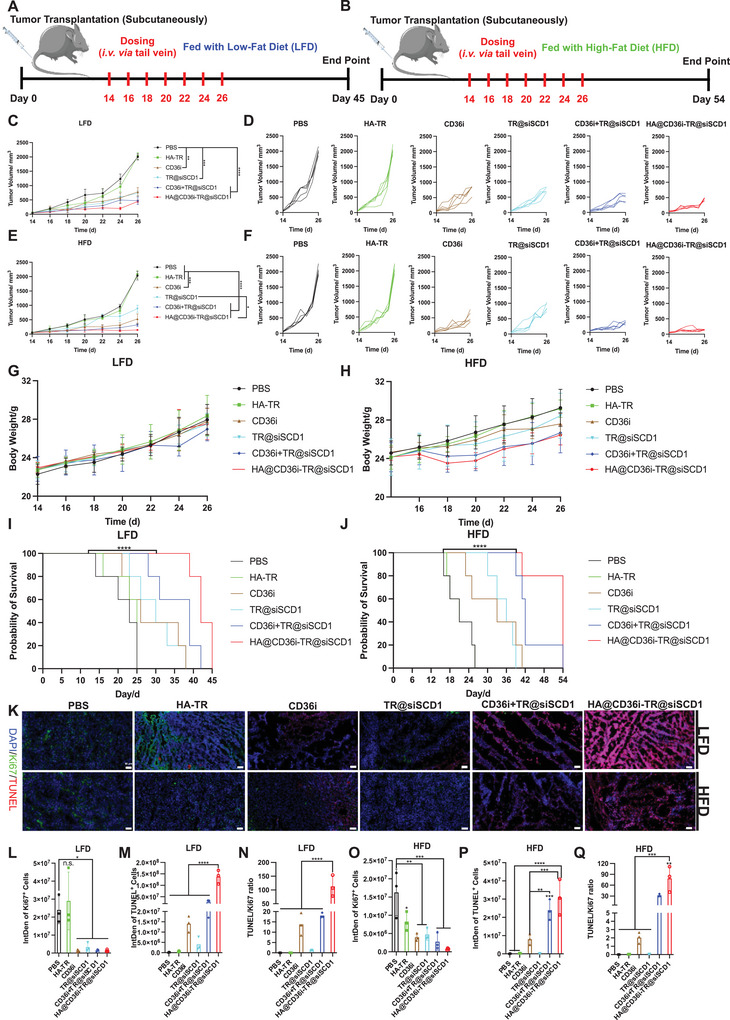
In vivo pharmacodynamics study of HA@CD36i‐TR@siSCD1. A,B) Schematic depicts the procedure of (A) LFD‐fed and (B) HFD‐fed RM‐1_Enz_ bearing C57BL/6J mouse model; C–F) Tumor volume curves of each group in (C,D) LFD‐fed model and (E, F) HFD‐fed model(n = 5, mean ± SD, one‐way ANOVA, ^*^ *p* < 0.05, ^**^ *p* < 0.01, ^***^ *p* < 0.001, ^****^ *p* < 0.0001); G,H) Body weight curves of each group in (G) LFD‐fed model and (H) HFD‐fed model (n = 5, mean ± SD); I,J) Survival curves of each group in (G) LFD‐fed model and (H) HFD‐fed model(n = 5, log‐rank analysis, ^****^ *p* < 0.0001); K) Typical immunofluorescence images of Ki67^+^ and TUNEL^+^ cells in each group in LFD‐fed and HFD‐fed models (bars = 50 µm); L–Q) Statistical analysis of Ki67^+^ cell signals, TUNEL^+^ cell signals and TUNEL/Ki67 ratios of each group in (L‐N) LFD‐fed and (O‐Q) HFD‐fed models (n = 3, mean ± SD, one‐way ANOVA, n.s.: no significance, ^*^ *p* < 0.05, ^**^ *p* < 0.01, ^***^ *p* < 0.001, ^****^ *p* < 0.0001).

To further investigate the efficacy of HA@CD36i‐TR@siSCD1 in vivo, C57BL/6J mice were transplanted with RM‐1_Enz_ cells and fed LFD or HFD, respectively, and the HA@CD36i‐TR@siSCD1 and other control groups were subjected to tail vein injection every 2 days from Day 14 to Day 26 (**Figure** **5**A,B). As shown in Figure 5C,D, during the dosing period of LFD‐fed mice, tumor growth was rapid in the PBS and HA‐TR groups, whereas tumor growth was significantly inhibited in both the monotherapy and combination groups (*p* < 0.01). The TR@siSCD1 group was superior to the CD36i group, and the combination groups were superior to the monotherapy groups. As shown in Figure 5E,F, during the dosing period of HFD‐fed mice, the PBS and HA‐TR groups presented rapid tumor growth, whereas the CD36i group and the combination groups presented significantly inhibited tumor growth (*p* < 0.001). In addition, there was almost no tumor growth in the HA@CD36i‐TR@siSCD1 group (*p* < 0.0001), while there was no significant difference in tumor growth between the TR@siSCD1 group and the PBS and HA‐TR groups. These results were in line with those in vitro. Under low‐fat conditions, both CD36i and TR@siSCD1 both showed strongly inhibited tumor growth. However, when lipids become the main energy source, endogenous inhibition of SCD1 is not enough to deplete the energy source of the tumor, and inhibition of lipid uptake in tumors significantly hinders tumor growth. Under high‐fat conditions, tumors are clearly more sensitive to endogenous + exogenous synergistic inhibition of lipid uptake and synthesis by CD36i + siSCD1. Since the energy source of LFD‐fed mice was mainly carbohydrates, the weight gain of each group was normal (Figure 5G). While the energy source of HFD‐fed mice was mainly lipids, weight gain was much slower in the combination groups than in the PBS and HA‐TR groups, suggesting that the HA@CD36i‐TR@siSCD1 nanosystem not only inhibited tumor growth but also prevented excessive obesity in HFD‐fed mice (Figure 5H). Notably, spinal metastasis was observed at the endpoint of the efficacy trials (Day 26) in the PBS and HA‐TR groups (data not shown).

Moreover, in LFD‐fed mice, compared with the PBS control group, the monotherapy and combination groups extended the median survival (MS) of the mice from 23 days to 42 days (PBS vs HA@CD36i‐TR@siSCD1, *p* < 0.0001) (Figure 5I). In HFD‐fed mice, the MS of the PBS and HA‐TR groups was only 21 days, whereas the MS of the CD36i and TR@siSCD1 groups was significantly prolonged (33 days and 36 days, respectively). Moreover, the MS of the CD36i+TR@siSCD1 group was 42 days, which was twice that of the PBS group, and the MS of the HA@CD36i+TR@siSCD1 group was 54 days, which was 2.6 times greater than that of the PBS group (Figure 5J).

Additionally, tumor tissues from each group were dissected, and immunofluorescence sections were made to observe the expression of nuclear‐associated antigen ki‐67 (Ki67) and terminal‐deoxynucleotidyl transferase mediated dUTP nick‐end labeling (TUNEL). As shown in Figure 5K–N, compared with those in the PBS group, the expression levels of Ki67 in the LFD‐fed mice were significantly lower in the monotherapy and combination groups (*p* < 0.05); and compared with those in the other control groups, the expression levels of TUNEL and TUNEL/Ki67 ratios were significantly greater in the HA@CD36i‐TR@siSCD1 group (*p* < 0.0001). Compared with that in the PBS group, Ki67 expression in the HFD‐fed mice was significantly lower (*p* < 0.01), whereas TUNEL expression in the TR@siSCD1 group was not significantly different from that in the PBS and HA‐TR groups. TUNEL expression (*p* < 0.0001) and the TUNEL/Ki67 ratio (*p* < 0.001) were significantly greater in the HA@CD36i‐TR@siSCD1 group than in the PBS group (Figure 5K,O–Q). These results indicated that HA@CD36i‐TR@siSCD1 significantly inhibited the proliferation and metastasis of tumor cells in vivo.

### 2.6 HA@CD36i‐TR@siSCD1 Enhanced Antitumor Immunity

**Figure 6:**
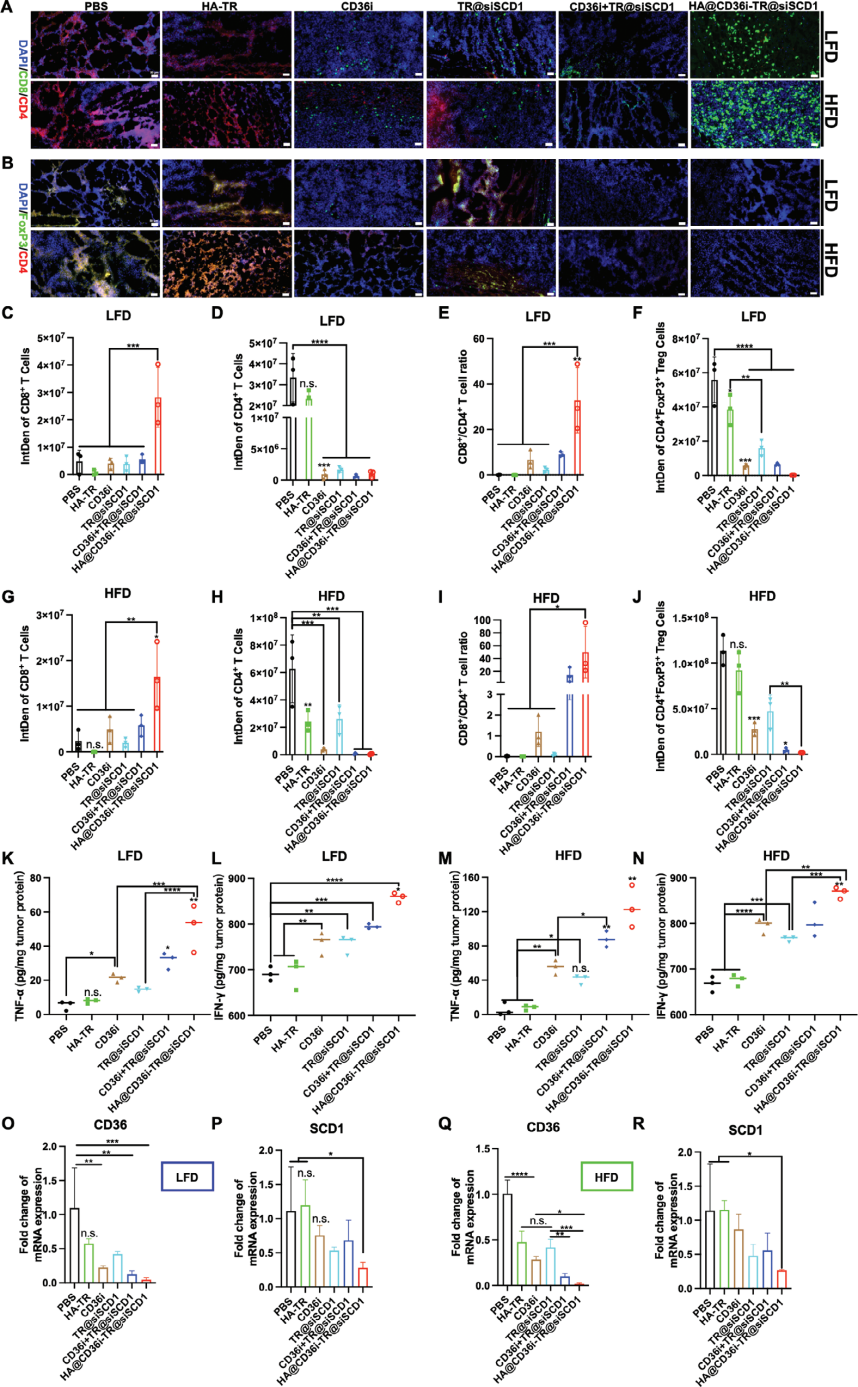
In vivo mechanism study. A,B) Typical immunofluorescence images of (A) CD8^+^ T cells and CD4^+^ T cells and (B) CD4^+^FoxP3^+^ Treg cells in each group in LFD‐fed and HFD‐fed models (bars = 50 µm); C–J) Statistical analysis of CD8^+^ T cell signals, CD4^+^ T cell signals, CD8^+^/CD4^+^ T cell ratios and CD4^+^FoxP3^+^ Treg cell signals of each group in (C‐F) LFD‐fed and (G‐J) HFD‐fed models (n = 3, mean ± SD, one‐way ANOVA, n.s.: no significance, ^*^ *p* < 0.05, ^**^ *p* < 0.01, ^***^ *p* < 0.001, ^****^ *p* < 0.0001); K–N) Intratumor TNF‐α and IFN‐γ levels of each group in (K, L) LFD‐fed and (M, N) HFD‐fed models (n = 3, mean ± SD, one‐way ANOVA, n.s.: no significance, ^*^ *p* < 0.05, ^**^ *p* < 0.01, ^***^ *p* < 0.001, ^****^ *p* < 0.0001); O–R) Intratumoral CD36 and SCD1 mRNA levels of each group in (O, P) LFD‐fed and (Q, R) HFD‐fed models (n = 3, mean ± SD, one‐way ANOVA, n.s.: no significance, ^*^ *p* < 0.05, ^**^ *p* < 0.01, ^***^ *p* < 0.001, ^****^ *p* < 0.0001).

Multiple studies have reported that inhibiting CD36 or SCD1 in prostate cancer or other tumors can promote antitumor immunity, increase CD8^+^‐T cell infiltration, and reduce the invasion of immunosuppressive cells such as Treg cells and TAMs.^[^ ^16^, ^17^, ^18^, ^19^, ^32^, ^33^ ^]^ As shown in **Figure** **6**A,C–E, in the PBS group and HA‐TR group of LFD‐fed mice, the tumor tissues were infiltrated with mainly CD4^+^ T cells, but the ratio of CD8^+^/CD4^+^ T cells gradually increased in the monotherapy and combination groups. In the HA@CD36i‐TR@siSCD1 group, the main type of infiltrating T cells changed to CD8^+^ T cells, and the CD8^+^/CD4^+^ T‐cell ratio increased significantly (*p* < 0.001). In HFD‐fed mice, the proportion of CD4^+^ T cells was greater than that of CD8^+^ T cells in the TR@siSCD1 group, possibly because TR@siSCD1 alone mainly targets tumor cells and cannot block lipid uptake by T cells. Excessive lipid uptake reportedly promotes the depletion of CD8^+^ T cells.^[^ ^18^ ^]^ When TR@siSCD1 was combined with CD36i, the proportion of CD8^+^ T cells increased significantly, and the CD8^+^/CD4^+^ T‐cell ratio in the HA@CD36i‐TR@siSCD1 group was greater than 50 (Figure 6A,G–I). Moreover, in LFD‐ or HFD‐fed mice, similar to those in the PBS and HA‐TR groups, many immunosuppressive CD4^+^FoxP3^+^ Treg cells infiltrated in the tumors of the TR@siSCD1 group, mainly because SCD1 had a limited regulatory effect on Treg cells, whereas Treg infiltration was significantly reduced in the CD36i administration groups (*p* < 0.001) (Figure 6B,F,J). In addition, the HA@CD36i‐TR@siSCD1 group presented significantly intratumoral levels of tumor necrosis factor‐alpha (TNF‐α) and interferon‐gamma (IFN‐γ) in both LFD‐ and HFD‐fed mice (*p* < 0.0001) (Figure 6K–N). These results suggested that HA@CD36i‐TR@siSCD1 can significantly increase the infiltration of intratumoral CD8^+^ T cells and reduce the infiltration of Treg cells, thereby enhancing antitumor immunity and turning “cold” tumors into “hot” tumors. Moreover, in line with the in vitro results, both CD36i and siSCD1 significantly inhibited their respective targets in LFD‐ or HFD‐fed mice. Compared with those in the PBS group, the intratumoral mRNA levels of CD36 and SCD1 in the HA@CD36i‐TR@siSCD1 group were significantly lower (*p* < 0.05) (Figure 6O–R). In addition, the expression of SCD1 protein in the HA@CD36i‐TR@siSCD1 group was significantly decreased (Figure S15, Supporting Information). These results suggest that the combined regulation of SCD1 and CD36 can reactivate antitumor immunity, thereby inhibiting the progression of refractory PCa.

### 2.7 HA@CD36i‐TR@siSCD1 Inhibited Lipid Accumulation in Tumors

**Figure 7:**
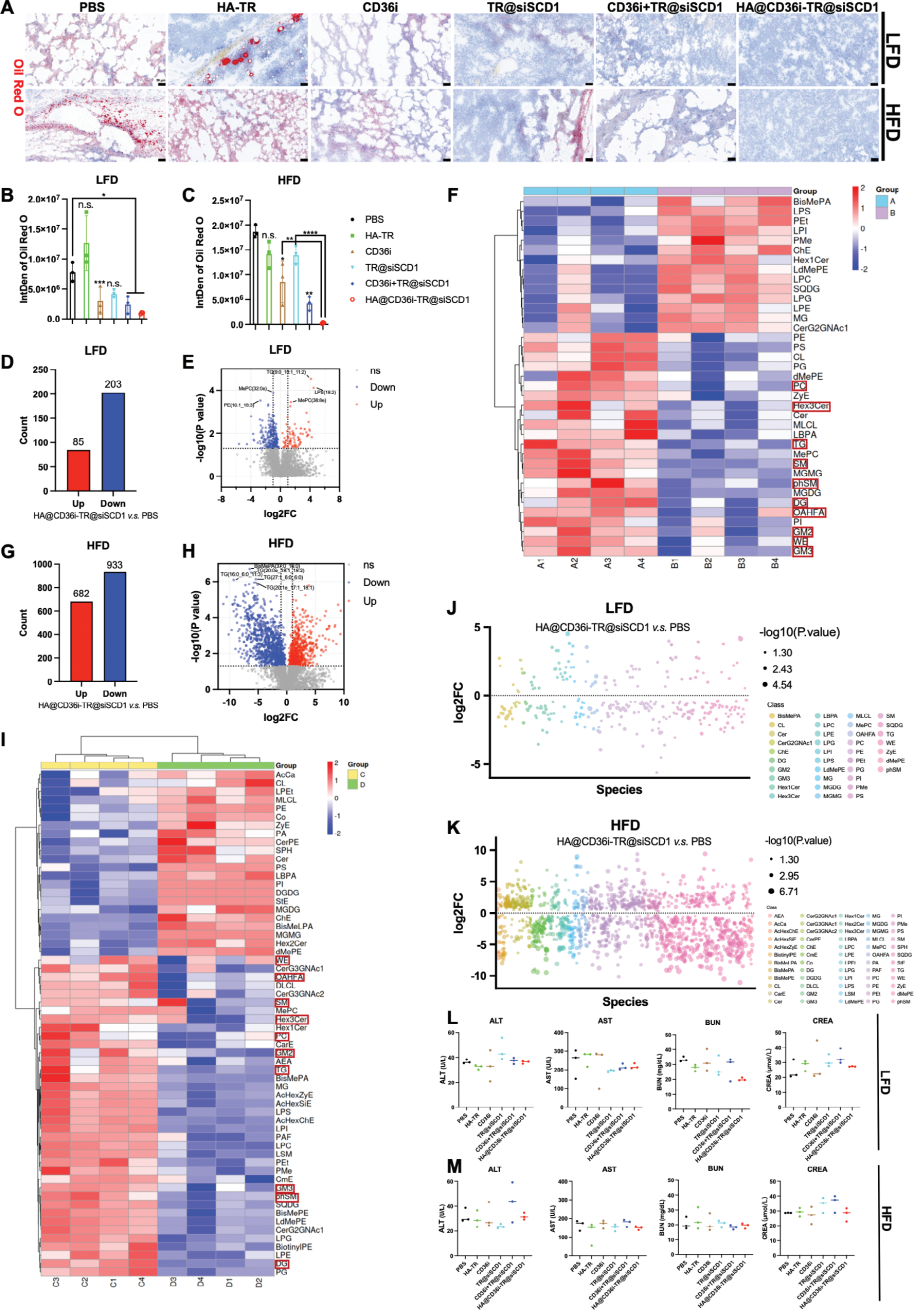
In vivo lipid metabolism and safety study. A) Typical oil red O staining images of each group in LFD‐fed and HFD‐fed models and B,C) Statistical analysis (bars = 50 µm, n = 3, mean ± SD, one‐way ANOVA, n.s.: no significance, ^*^ *p* < 0.05, ^**^ *p* < 0.01, ^***^ *p* < 0.001, ^****^ *p* < 0.0001); D–I) Statistic of differently expressed lipids, volcano plot data, and differential lipid classification heatmap in (D‐F) LFD‐fed model and (G‐I) HFD‐fed model, HA@CD36i‐TR@siSCD1 group versus PBS group (Group A: PBS group fed LFD, Group B: HA@CD36i‐TR@siSCD1 group fed LFD, Group C: PBS group fed HFD, Group D: HA@CD36i‐TR@siSCD1 group fed HFD, n = 4, *p* value < 0.05); J,K) Differential lipid bubble map in (J) LFD‐fed model and (K) HFD‐fed model, HA@CD36i‐TR@siSCD1 group versus PBS group (n = 4, *p* value < 0.05, values were expressed as log2 (FC)); L,M) Plasma ALT, AST, BUN and CREA levels of each group in (L) LFD‐fed model and (M) HFD‐fed model (n = 3).

As reported in the literature, the inhibition of CD36 can inhibit cellular lipid uptake, and the inhibition of SCD1 can inhibit downstream lipid synthesis.^[^ ^11^, ^18^ ^]^ First, we examined lipid accumulation in the tumor tissue of each group of LFD‐ and HFD‐fed mice. As shown in **Figure** **7**A, there were abundant lipid droplets in both the PBS group and the HA‐TR group, while lipid accumulation in both the monotherapy groups and the combination groups was reduced to varying degrees, and almost no lipid accumulation was observed in the HA@CD36i‐TR@siSCD1 group. Compared with LFD‐fed mice, although HFD‐fed mice were not sensitive to siSCD1 monotherapy, they were more sensitive to the CD36i+TR@siSCD1 and HA@CD36i‐TR@siSCD1 combination therapies, and lipid accumulation was significantly lower in the HA@CD36i‐TR@siSCD1 group than in the PBS group (*p* < 0.0001), which was in line with the in vitro findings (Figure 7B,C).

After that, the tumors from each group were collected for nontargeted lipidomic analysis. Multivariate statistical analysis, including orthogonal partial least squares‐discriminant analysis (OPLS‐DA), principal component analysis (PCA) and patical least squares‐discriminant analysis (PLS‐DA), revealed significant differences between the HA@CD36i‐TR@siSCD1 group and the PBS group in both LFD‐fed mice and HFD‐fed mice (Figure S16, Supplementary Material 2–4, Supporting Information). In LFD‐fed mice, 85 lipids were upregulated and 203 lipids were downregulated in the HA@CD36i‐TR@siSCD1 group compared with those in the PBS group (Figure 7D,E; Supplementary Material 5, Supporting Information), whereas in HFD‐fed mice, 682 lipids were upregulated and 933 lipids were downregulated (Figure 7G,H; Supplementary Material 6, Supporting Information). As shown in Figure 7F,I, sphingomyelin (SM), phytosphingosine (phSM), gangliosides GM2 and GM3, hexosylceramide Hex3Cer, triglyceride (TG), diglyceride (DG), phosphatidylcholine (PC), (O‐acyl)‐1‐hydroxy fatty acid (OAHFA) and wax ester (WE) were decreased in both models. The differential lipid bubble map further demonstrated the regulatory effect of HA@CD36i‐TR@siSCD1 on tumor lipid metabolism in tumors, and the levels of most lipids tended to decrease (Figure 7J,K).

In addition, serum samples from the two models were collected for the detection of aspartate transaminase (AST), alanine transaminase (ALT), blood urea nitrogen (BUN) and creatinine (CREA) levels, and the results revealed that there were no significant differences in each index among the groups, indicating that HA@CD36i‐TR@siSCD1 had no obvious hepatorenal toxicity (Figure 7L,M). In addition, the HE staining results of the heart, liver, spleen, lung and kidney also revealed no significant damage to major organs in any of the groups, and the trend of tumor damage was consistent with the results of the in vivo efficacy experiments (Figure S17, Supporting Information). These results indicated that HA@CD36i‐TR@siSCD1 was biocompatible in vivo and had no obvious toxicity to major organs.

## 3 Discussion

Drug resistance is a major obstacle to the treatment of advanced PCa, and lipid metabolism plays an important role in the progression and drug resistance of PCa.^[^ ^7^, ^8^, ^14^, ^34^, ^35^ ^]^ According to the Warburg effect proposed by Otto Warburg, glycolysis is generally the main energy source of tumor cells, whereas PCa is also highly dependent on lipid uptake and synthesis, and the accumulation of lipids is conducive to the storage of carbon and energy in PCa to promote its progression and drug resistance.^[^ ^36^, ^37^, ^38^ ^]^ More interestingly, in the absence of adequate lipid synthesis, glycolysis cannot provide sufficient energy to sustain the growth of PCa cells.^[^ ^8^, ^39^ ^]^ Hence, regulating lipid metabolism and eliminating important energy sources in PCa is a promising treatment for refractory PCa.

CD36 is an important membrane channel protein for the cellular uptake of FAs and plays important roles in tumor metabolism, immunity, drug resistance, progression and metastasis,^[^ ^15^, ^16^, ^17^, ^18^, ^19^ ^]^ and CD36 is highly expressed in Enz‐resistant PCa cells (Figure S7, Supporting Information). In addition to blocking the uptake of exogenous fatty acids, suppressing cellular lipid synthesis is crucial for tumor growth. SCD1 is the rate‐limiting enzyme of MUFAs and is closely related to the accumulation of lipid droplets, and the inhibition of SCD1 can delay tumor progression and drug resistance.^[^ ^11^, ^12^, ^13^, ^14^ ^]^

In this study, we selected a combination of a CD36 inhibitor (sulfosuccinimidyl oleate sodium, CD36i) and SCD1 siRNA (siSCD1) for the treatment of Enz‐resistant PCa. Given that CD36i mainly acts on CD36 on the cell membrane of tumor cells and immune cells, and that siSCD1 needs to enter tumor cells to perform gene silencing, we designed a multiresponsive core‐shell structured nanosystem, HA@CD36i‐TR@siSCD1. The HA gel shell can actively target CD44 expressed on tumor cells and depolymerize in response to the acidic TME and HAase, while TR@siSCD1 micelles can target PCa cells with the guidance of the RGD peptide and release siSCD1 in response to intracellular GSH and the proton sponge effect^[^ ^2^, ^22^, ^27^, ^31^ ^]^ (Figures 1H and 2A). CD36i and siSCD1 synergistically suppressed the proliferation, invasion, and metastasis of Enz‐resistant PCa cells and tumoroids. Notably, when OA, the downstream product of SCD1, was supplemented in vitro, the anticancer effect of siSCD1 was significantly reduced, but PCa cells were still sensitive to combined CD36i alone and CD36i + siSCD1 therapy. This is mainly because the endogenous OA inhibited by siSCD1 was supplemented by exogenous OA, while CD36i can block the uptake of exogenous OA by PCa cells, so PCa cells maintain their sensitivity to the CD36i, with significantly increased anti‐proliferative, metastic and progressive effects (Figures 3, 4; Figures S8–12, and Tables S1–S5, Supporting Information). In vivo, the mice were fed a LFD or a HFD, respectively. Similarly, HFD‐fed mice were insensitive to the TR@siSCD1 monotherapy, but were significantly sensitive to the CD36i + siSCD1 combination therapy. HA@CD36i‐TR@siSCD1 significantly prolonged the survival of HFD‐fed mice (median survival, HA@CD36i‐TR@siSCD1 vs PBS, 54 days vs 21 days) and prevented them from gaining too much weight (Figure 5; Table S6, Supporting Information), with good biocompatibility and safety (Figure 7L,M; Figure S14, Supporting Information). In some tumors that rely primarily on glycolysis for energy, researchers have attempted to replace carbohydrates with a ketogenic diet. These tumors cannot utilize ketogenesis, thereby depriving the energy source of tumors and inhibiting their growth and metastasis.^[^ ^40^ ^]^ However, in PCa, lipids are important energy sources.^[^ ^7^ ^]^ Thus, in HFD‐fed mice, the inhibition of lipid metabolism, especially lipid uptake, can directly cut off the energy source of PCa, and the inhibition of lipid synthesis can further deplete its energy source. This may be the reason that HFD‐fed mice are more sensitive to HA@CD36i‐TR@siSCD1 treatment.

Moreover, after HA@CD36i‐TR@siSCD1 treatment, we observed that in both LFD‐fed and HFD‐fed mice, the intratumoral CD8^+^/CD4^+^ T‐cell ratios increased significantly, with decreased Treg cells and increased TNF‐α and IFN‐γ levels, indicating that PCa transformed from a “cold” tumor to a “hot” tumor (Figure 6). CD36 plays an important role in tumor immunity, and the overexpression of CD36 in tumors can promote Treg cell differentiation and CD8^+^ T‐cell dysfunction, thereby reducing the secretion of the tumor killer factors TNF‐α and IFN‐γ.^[^ ^17^ ^]^ In addition, it is reported that overexpressed SCD1 can attenuate the antitumor CD8^+^ T cell response, and SCD1 is essential for the ability of Treg cells to suppress conventional T cells in multiple tumors, leading to the progression of tumor growth and metastasis.^[^ ^41^, ^42^, ^43^, ^44^ ^]^ In tumors, as increased lipid metabolism has become a hallmark of tumors and is closely related to their malignant progression, Treg cells are dominant in tumors and rely on lipid accumulation, which is facilitated by upregulated SCD1‐mediated lipid synthesis.^[^ ^45^ ^]^ However, in autoimmune diseases, SCD1 seems to play the opposite role. A recent study revealed that OA, a downstream product catalyzed by SCD1, is a key suppressor of superfluous peripheral Treg cell generation under physiological conditions, and that *Scd1* deficiency in thymic epithelial cells results in elevated levels of peripheral Treg cells through the regulation of OA availability in early developing thymocytes, but the number of CD4^+^FoxP3^+^ Treg cells is not affected.^[^ ^46^ ^]^ SCD1 has also been shown to enhance Treg differentiation in autoimmune disease models.^[^ ^47^ ^]^ SCD1 plays a regulatory role in the immune system, but it is clear that SCD1 has gone out of control in tumors. Generally, CD36i and siSCD1 synergistically enhance antitumor immunity in the treatment of refractory PCa.

In addition, nontargeted lipidomics revealed an overall downregulation of lipid metabolism in both LFD‐fed and HFD‐fed mice, and SM, phSM, GM2, GM3, Hex3Cer, TG, DG, PC, OAHFA and WE were downregulated in both models. In addition, almost no accumulation of lipid droplets was observed in HA@CD36i‐TR@siSCD1‐treated mice (Figure 7A–K). In the tumors of HA@CD36i‐TR@siSCD1‐treated mice, lipid accumulation was significantly decreased, and the uptake of TG and DG was significantly decreased. Moreover, SM in the cell membrane has been reported to be closely related to immune escape, and tumorigenesis initiation and progression, and the inhibition of antitumor immunity may be due to the activation and differentiation of Treg cells promoted by SM metabolism.^[^ ^48^, ^49^ ^]^ In contrast, phSM inhibited tumor progression in several tumor studies.^[^ ^50^, ^51^, ^52^ ^]^ The ganglioside GM2 mediates tumor migration, while GM3, which is overexpressed on tumor cells, is defined as a tumor‐associated carbohydrate antigen and is used for immunotherapy.^[^ ^53^, ^54^ ^]^ In addition, the decrease in PC may be attributed to PC hydrolysis mediated by the inhibition of SCD1,^[^ ^42^ ^]^ but the relationships among OAHFA, WE and tumors have rarely been reported. Overall, these downregulated lipids were associated with enhanced antitumor immunity and inhibition of tumor progression.

## 4 Conclusion

In summary, we are the first to utilize CD36i in combination with siSCD1 to block lipid uptake and synthesis in refractory PCa, and designed a multiresponsive targeted drug delivery system, HA@CD36i‐TR@siSCD1. HA@CD36i‐TR@siSCD1 can first accumulate at tumor sites by targeting CD44 and then depolymerize in response to the acidic TME and HAase and release CD36i to regulate the tumor microenvironment and lipid uptake. TR@siSCD1 can then target tumor cells via the RGD peptide segment and release siSCD1 in the cytoplasm through the proton sponge effect and the GSH response to downregulate SCD1 and its downstream products. In this study, the in vitro and in vivo experiments revealed that the HA‐TR vector has good gene compression and transfection efficiency as well as biocompatibility, and HA@CD36i‐TR@siSCD1 has a good synergistic effect, which can significantly inhibit the proliferation, invasion and metastasis of refractory PCa without significant toxicity. In addition, although PCa was insensitive to siSCD1 monotherapy under high‐fat conditions, it was more sensitive to CD36i + siSCD1 combination therapy than was low‐fat conditions. The HA@CD36i‐TR@siSCD1 group presented the strongest tumor suppression and lipid metabolism regulation ability, and almost no lipid accumulation was observed in the tumor tissue of the HA@CD36i‐TR@siSCD1 group. Moreover, HA@CD36i‐TR@siSCD1 had a potent immune‐enhancing effect, increasing the proportion of CD8^+^ T cells, decreasing the number of CD4^+^FoxP3^+^ Treg cells, promoting the release of the tumor killer factors TNF‐α and IFN‐γ, and transforming refractory PCa from “cold” tumors to “hot” tumors. Therefore, this study is expected to provide a combination therapy based on lipid metabolism regulation for patients with refractory PCa, especially those with a HFD.

## Conflict of Interest

The authors declare no conflict of interest.

## Supporting information



Supporting Information

## Data Availability

The data that support the findings of this study are available from the corresponding author upon reasonable request.
