# Supplementary material for: Combined Blockade of Lipid Uptake and Synthesis by CD36 Inhibitor and SCD1 siRNA Is Beneficial for the Treatment of Refractory Prostate Cancer
Source: Adv Sci (Weinh). 2024 Dec 30;12(8):2412244. doi: 10.1002/advs.202412244 (PMC11848597; doi:10.1002/advs.202412244)
Supplement: Supplementary file 1 — Supporting Information [file ADVS-12-2412244-s001.docx]

Supporting Information

Combined Blockade of Lipid Uptake and Synthesis by CD36 Inhibitor and SCD1 siRNA is Beneficial for the Treatment of Refractory Prostate Cancer

Jiyuan Chen, Xiaoyan Yu, Gang Yang, Xueying Chen, Chunai Gong, Lu Han, Yujie Wang, Rong Wang, Lei Wang, and Yongfang Yuan

J. Chen, X. Yu, G. Yang, C. Gong, L. Han, Y. Wang, R. Wang, L. Wang, Y. Yuan

Department of Pharmacy, Shanghai Ninth People’s Hospital, Shanghai Jiao Tong University School of Medicine, Shanghai 200011, P. R. China
E-mail: nmxyyf@126.com

X. Chen
Department of Stomatology, The Sixth Affiliated Hospital of Sun Yat-Sen University, Guangzhou 510655, P.R. China

**This file includes:**

Materials and methods, Figure S1-S17 and Table S1-S6.

**Materials and Methods**

***Materials*.** RMPI 1640 medium, penicillin/streptomycin and fetal bovine serum (FBS) were purchased from Gibco, USA. Phosphate buffered saline (PBS) was purchased from BasalMedia, China. (Gibco, USA), TrypLE^TM^ Express (Phenol Red Free) (Gibco, USA), Matrigel® Matrix was purchased from Corning, USA. CellTiter-Glo® 3D Cell Viability Assay were purchased from Promega, USA. Hyaluronate methacrylate (HAMA), lithium Phenyl-(2,4,6-trimethylbenzoyl)-phosphinate (LAP) and hyaluronidase (HAase) were purchased from StemEasy, China. N,N’-methylenebis(acrylamide) (MBA) were purchased from Rhawn, China. TR peptide (LA-rkkrrqrrrHlKYDGR, where LA is lipoic acid, and lowercase letter indicates that the amino acid is a D-type amino acid) was synthesized by Zhejiang Ontores Biotechnologies Co., Ltd., China and verified by Beijing Biotech-Pack Scientific Co., Ltd., China. Dithiothreitol (DTT) was purchased from Sangon Biotech, China. CD36 inhibitor sulfosuccinimidyl oleate (CD36i) were purchased from MCE, USA. Human SCD1 siRNA (siSCD1) (sense: GCACAUCAACUUCACCACATT, antisense: UGUGGUGAAGUUGAUGUGCTT) and mouse siSCD1 (sense: CUUUAAUCAACCCAAGAAATT, anti-sense: UUUCUUGGGUUGAUUAAAGGC), scramble siRNA (NC siRNA), FAM-labeled scramble siRNA (siFAM), Cy3-labeled scramble siRNA (siCy3) and plasmid of enhanced green fluorescent protein (pEGFP) were purchased from GenePharma, China. 50× TAE (Tris/Acetic Acid/EDTA) Buffer (Sangon Biotech, China), agarose (Biowest, Spain), and Gelred (Biotium, USA), and diethyl pyrocarbonate (DEPC) water (Fushenbio, China) were purchased for aragose gel electrophoresis and gene transfection studies. Cell Counting Kit-8 (CCK-8) and Annexin V-FITC/PI Cell Apoptosis Kit were from Beyotime Biotech, China. Fluorescent substances DAPI (λex/λem = 358/461 nm) (Yeasen Bio, China), Lysotracker Red (λex/λem = 577/590 nm) (Beyotime Biotech, China), MitoTracker Green (λex/λem = 490/516 nm) (Beyotime Biotech, China) Nile Red (Nile, λex/λem = 552/636 nm) (Yuanyebio, China) and 1,1-dioctadecyl-3,3,3,3-tetramethylindotricarbocyaine iodide (DiR, λex/λem = 748/780 nm) (Biotium, USA) were used for fluorescence quantitative analysis and/or imaging analysis. Ultrapure water was prepared by a Milli-Q ultrapure water system (Merck, Germany). Oil red O and oleic acid (OA) were purchased from Solarbio, China. XTHF60 high-fat fodder (60% fat, 20% carbohydrate, 20% protein) was purchased from Jiangsu Xietong Pharmaceutical Bio-engineering Co., Ltd., China.

***Cell lines*.** The human prostate cancer cell line C4-2B and PC-3, human embryonic kidney cell line HEK-293T and mouse prostate cancer cell line RM-1 were provided by Shanghai Cell Bank, Chinese Academy of Sciences (CAS, China), and incubated in RPMI 1640 medium supplemented with 10% FBS and 1% penicillin/streptomycin. Enzalutamide-resistant cell line C4-2B_Enz_, PC-3_Enz_ and RM-1_Enz_ were obtained according to the reported method ^[1,2]^. Briefly, enzalutamide was dissolved in vehicle (10% DMSO, 45% polyethylene glycol 400, and 45% PBS) as a mother liquor and diluted to a specific concentration with complete medium. The enzalutamide-sensitive PC-3 and RM-1 cells were cultured at 20 μM enzalutamide for at least 3 months and the developed PC-3_Enz_ and RM-1_Enz_ cells were maintained in media with 20 μM enzalutamide.

***Animals*.** C57BL/6J mice (male, 4-6 weeks old, 18-22 g) were purchased from Shanghai Jiesijie Laboratory Animal Co., Ltd. (experimental animal license number was SCXK (Shanghai) 2023-0004) and Gempharmatech Co., Ltd. (experimental animal license number was SCXK (Jiangsu) 2023-0009). All the mice were randomly divided into two groups. One group was fed a low-fat diet (LFD) and one group fed a high-fat diet (HFD) (Jiangsu Xietong Pharmaceutical Bio-engineering Co., Ltd., China). All animal experiments were in line with the ethical requirements of the Laboratory Animal Ethics Committee in Ninth People’s Hospital Affiliated to Shanghai Jiao Tong University School of Medicine, with approved ethical review numbers SH9H-2022-A925-1.

***Evaluation of drug resistance prostate cancer cell line*.** The C4-2B_Enz_ cell line has been constructed according to the former method ^[1,2]^. To confirm the establishment of PC-3_Enz_ and RM-1_Enz_ cells, the IC_50_ values of enzalutamide in PC-3/PC-3_Enz_ and RM-1/RM-1_Enz_ cells were calculated by a CCK-8 kit, with an enzalutamide concentration gradient of 0-200 μg/mL (Spark^®^ multifunctional microplate reader, Tecan, Swiss). Finally, a cell clone formation experiment was applied to further verify the establishment of PC-3_Enz_ and RM-1_Enz_ cells. When cells grew to logarithmic growth phase, PC-3/PC-3_Enz_ and RM-1/RM-1_Enz_ cells were seeded in 6-well plates in triplicate with agarose matrix at a density of 50 cells/well and cultured in RMPI 1640 complete medium with or without 40 μM enzalutamide for 2 weeks. The culture medium was replaced with fresh medium every 4 days. After 2 weeks, the cell clones were stained with 0.1% crystal violet, photographed and observed with a fluorescence microscope (Olympus IX73, Japan) in bright field.

***Preparation and characterization of TR peptide*.** TR peptide (LA-rkkrrqrrrHlKYDGR, where LA is lipoic acid, and lowercase letter indicates that the amino acid is a D-type amino acid) was synthesized using the Fmoc-solid-phase peptide synthesis method by Zhejiang Ontores Biotechnologies Co., Ltd., China, and the product was purified and characterized by High Performance Liquid Chromatography (HPLC) and Mass Spectrometer (MS). The HPLC (Waters 2695, USA) was equipped with a column (agilent ZORBAX 300SB-C18 5 μm, 4.6 × 250 mm), mobile phase: 0→10 min, phase A (0.1% trifluoroacetic acid in H_2_O): 100%→0%, phase B (acetonitrile): 0%→100%, flow rate: 1 mL/min, wavelength: 220 nm; whereas the MS was equipped with a column (COSMOSIL 5C18-MS-II, 4.6 × 250 mm) at 30℃. Wavelength: 220 nm, diluent: 5% acetonitrile, preparation: 0.3 mg/mL, injection volume: 10 μL, mobile phase: 0→20 min, phase A (0.1% trifluoroacetic acid in H_2_O): 88%→68%, phase B (acetonitrile): 12%→32%, flow rate: 1 mL/min.

Moreover, the sequence of rkkrrqrrrHlKYDGR peptide was qualified by LC-MS/MS. Sample pretreatment: the rkkrrqrrrHlKYDGR peptide was dissolved (1 mg/mL) and added with 10 mM DTT, and reduced it in a water bath at 56℃ for 1 h. Next, 3-indoleacetic acid (IAA) solution with a final concentration of 50 mM was added, and the reaction was carried out for 40 min away from light. Then, the sample was desalted with a self-filling desalting column and dried in a 45℃ vacuum centrifugal concentrator. Finally, the sample was redissolved in 500 μL sample solution (0.1% formic acid, 2% acetonitrile), fully oscillated vortex, 13200 rpm, centrifuge at 4℃ for 10 min, supernatant was transferred to the upper sample tube, waiting for mass spectrometry analysis. LC-MS/MS detection: i. Conditions for capillary liquid chromatography (Ultimate 3000, Thermo Fisher, Scientific, USA): precolumn: 300 μm × 5 mm, packed with Acclaim PepMap RPLC C18, 5μm, 100Å; analysis column: 75 μm × 150 mm, packed with Acclaim PepMap RPLC C18, 3μm, 100 Å; mobile phase: phase A (0.1% formic acid, 2% acetonitrile): 0→1 min: 96%-92%, 1→21 min: 92%-72%, 21→26 min: 72%-60%, 26→27 min: 60%-10%, 27→37 min: 10%, phase B (0.1% formic acid, 80% acetonitrile): 0→1 min: 4%-8%, 1→21 min: 8%-28%, 21→26 min: 28%-40%, 26→27 min: 40%-90%, 27→37 min: 90%; flow rate: 600 nL/min; loading quantity: 5μL. ii. Conditions for MS (Q Exactive™ Hybrid Quadrupole-Orbitrap™ Mass Spectrometer, Thermo Fisher Scientific, USA): Primary mass spectrum parameters: resolution: 70,000, AGC target: 3e6, maximum injection time: 60 ms, scan range: 300 to 1800 m/z; Secondary mass spectrum parameters: resolution: 17,500, AGC target: 5e4, maximum injection time: 15 ms, TopN: 5, scan range: 200 to 2000 m/z.

***Preparation of TR micelles and TR@siSCD1*.** Ten milligrams TR peptide and 1 mg cystine were codiluted in 5 mL ultrapure water and stirred for at least 8 h in a sealed Eppendorf tube. Then, the self-assembly TR micelles were purified in a 10,000 Da dialysis bag for 24 h. The critical micelle concentration (CMC) of TR micelles was tested by using a potentiometric analysis method.TR micelles and siSCD1 (N/P ratio = 50) were cocultured in room temperature for 30 min to obtain TR@siSCD1. TR micelles and TR@siSCD1 was characterized with a dynamic light scattering (DLS) system (Malvern Zetasizer Pro, Nano-ZS90, UK). TR micelles was witnessed under a transmission electronic microscope (TEM) (JEM-2100, Japan).

***Preparation of HA-TR and HA@CD36i-TR@siSCD1*.** To prepare HA@CD36i-TR@siSCD1, 1 mg CD36i, 1 mg HAMA, 1 mg LAP and 1 mg MBA were dissolved in 1 mL TR@siSCD1 micelles (TR: 1 mg/mL), stirred continuously under 405 nm blue light at room temperature for 8 h. Then, HA@CD36i-TR@siSCD1 was purified by ultrafiltration with 100 k MWCO ultrafiltration spin columns (Millipore, USA). HA-TR was prepared in the same way as above. The drug encapsulation efficiency (EE) and drug loading (DL) rates of drugs were calculated according to the following formulas.

$DL\left( \% \right)= \frac{Mass of Drug in the Nanoparticles}{Total Mass of Nanoparticles} \times100\%$ (1)

$EE\left( \% \right)\frac{Mass of Drug in the Nanoparticles}{Total Mass of Drug} \times100\%$ (2)

***Stability investigation*.** To investigate the stability of TR micelles and HA-TR, TR micelles and HA@BSA-TR were dispersed in PBS buffer at a concentration of 1 mg/mL and stored at 4 ℃, the 1-month stability of TR micelles and HA@BSA-TA was measured by DLS.

***Drug release of siRNA*.** To study the *in vitro* release behavior of siRNA-loaded HA-TR, 1 mL of HA-TR@siCy3 (siCy3: 20 μg/mL) was placed in a 50 kD (Yibo Biological, China) dialysis bag, immersed in 50 mL of PBS buffer (pH 6.5 or 7.4) with constant stirring, added with or without 50 mM DTT or 1 mg/mL HAase. At 0, 0.5, 1, 2, 4, 8, 12 and 24 h, 1 mL of liquid was taken (replenished an equal volume of buffer) and measured with a fluorescence microplate reader (VirioSkan Flash microplate reader, Thermo, USA).

***Gene compression assay*.** To confirm that siRNA could be compressed by TR micelles, TR micelles were coincubated with pEGFP at a N/P ratio of 0:1, 1:1, 2:1, 4:1, 6:1, 10:1, 15:1 and 20:1 (pEGFP: 1 μg/well) for 30 min under RT and loaded with 5 μL GelRed for agarose gel electrophoresis study (Beijing Liuyi Biotechnology Co., Ltd., China), observing under 280 nm ultraviolet light. In addition, to analyze the redox sensitivity of TR micelles, different N/P ratios of TR@pEGFP were mixed with 50 mM DTT for agarose gel electrophoresis and witnessed under 280 nm ultraviolet light.

***Gene transfection assay*.** To evaluate the gene transfection efficiency of TR micelles, pEGFP were used as a model drug, and traditional cationic materials PEI was used as controls. HEK-293T cells were seeded in 24-well plates (10^5^ cells/well) overnight. HEK-293 cells were treated with TR@pEGFP at N/P ratios of 20:1, 30:1, 40:1, and 50:1 for 24 h and detected by a fluorescence microscopy. Additionally, to assess the toxicity of the vehicles and materials *in vitro*, a CCK-8 kit was used for the cell viability study. HEK-293T cells were seeded in 96-well plates at a concentration of 5 × 10^3^ cells/well and incubated overnight. Then, HEK-293T cells were treated with PEI, TR micelles, and HA-TR at concentrations of 0, 9.375, 18.75, 37.5, 75, 150, 300, 600, and 1200 μg/mL for 24 h respectively, and analyzed with a CCK-8 kit.

***Cellular uptake analysis*.** To evaluate the cellular uptake ability of prostate cancer cells to TR micelles and HA-TR nanoparticles, fluorescent substance Nile red (Nile) and FAM-labeled siRNA (siFAM) were used as model drugs. C4-2B_Enz_ or RM-1_Enz_ cells were seeded in 24-well plates at a concentration of 10^5^ cells/well. After 24 h, C4-2B_Enz_ or RM-1_Enz_ cells were treated with Nile, TR@Nile, or HA-TR@Nile (Nile: 100 ng/well). After 1 h of coincubation, all the groups were witnessed with a fluorescence microscope (Olympus IX73, Japan). Meanwhile, Moreover, C4-2B_Enz_ or RM-1_Enz_ cells were seeded in 24-well plates at a concentration of 10^5^ cells/well for 24 h and treated with PBS, siFAM, TR@siFAM, HA-TR@siFAM (siFAM: 500 ng/well). After 4 h of coincubation, all the groups were tested by a CytoFLEX^®^ flow cytometer (Beckman Coulter, USA).

***Lysosome escape assay*.** To confirm that HA-TR@siFAM could be delivered into cells without being degraded by lysosomes, C4-2B_Enz_ cells were treated with HA-TR@siFAM for 0-4 h, and after 1 h and 4 h of coincubation, cells were labeled with LysoTracker Red for 30 min, respectively. Then, the cells were fixed with 4% paraformaldehyde and stained with DAPI, observed and photographed under a confocal laser scanning microscopy (ZEISS LSM 800, German).

***Cell viability assay*.** To verify the *in vitro* effect of HA@CD36i-TR@siSCD1, C4-2B_Enz_ or RM-1_Enz_ cells were seeded in 96-well plates at a concentration of 5 × 10^3^ cells/well, with or without 500 μM OA. After 24 h, cells were treated with HA@CD36i-TR@siSCD1and other control groups, respectively at a siRNA concentration gradients of 0, 15.625, 31.25, 62.5, 125, 250, 500, 1000, and 2000 nM as well as a CD36i concentration gradients of 0, 1.5625, 3.125, 6.25, 12.5, 25, 50, 100, and 200 μg/mL, respectively. After 24 h of coincubation, the cell viability of each group was tested by a CCK-8 kit with a Plate reader Infinite^®^ M200Pro (Tecan, Swiss) or a Spark^®^ multifunctional microplate reader (Tecan, Swiss). Moreover, the combination index (CI) values of CD36i + TR@siSCD1 and HA@CD36i-TR@siSCD1 were calculated by CompuSyn software.

***Cell apoptosis study*.** C4-2B_Enz_ or RM-1_Enz_ cells were seeded in 24-well plates at a concentration of 10^5^ cells/well, with or without 500 μM OA. After 24 h, cells were treated with HA@CD36i-TR@siSCD1and other control groups, respectively (CD36i: 20 μg/mL, siSCD1: 100 nM). After 24 h of incubation, the apoptosis cells were detected and analyzed with a CytoFLEX^®^ flow cytometry.

***Anti-metastasis and anti-invasion assays*.** Transwell plates were used to assess the antimetastatic and anti-invasive abilities of HA@CD36i-TR@siSCD1. C4-2B_Enz_ cells were seeded in the upper chamber of an 8-μm Transwell plate at a concentration of 10^5^ cells/well and incubated with 100 μL FBS-free RPMI 1640 medium. For antimetastasis study or anti-invasion study, the upper chamber was coated without or with Matrigel-coated Transwell inserts. Meanwhile, 800 μL RPMI 1640 medium + 20% FBS was added to the lower chamber of Transwell. The cells were treated with HA@CD36i-TR@siSCD1 and other control groups, respectively (CD36i: 20 μg/mL, siSCD1: 100 nM). After 24 h or 48 h of incubation, the migrated or invaded cells were fixed and stained with 0.1% crystal violet for antimigration study or anti-invasion study, respectively. Three fields of the migrated or invaded cells were selected randomly and photographed and all the images were analyzed with ImageJ software.

***Lipid uptake experiment*.** C4-2B_Enz_ or RM-1_Enz_ cells were seeded in 24-well plates at a concentration of 10^5^ cells/well, with or without 500 μM OA. After 24 h, cells were treated with HA@CD36i-TR@siSCD1and other control groups, respectively (CD36i: 20 μg/mL, siSCD1: 100 nM). After 24 h of incubation, the cells were dyed with oil red and witnessed under bright field with a fluorescence microscope (Olympus CKX53, Japan) to assess cellular lipid accumulation in each group.

***Tumoroid study*.** Tumor spheroids were prepared as previous reported ^[1]^. C4-2B_Enz_ tumor spheroids were treated with free Nile, free siFAM, TR@siFAM or HA@Nile-TR@siFAM, respectively (Nile: 100 ng/mL, siFAM: 1 μg/mL). After 4 h of incubation, all the tumoroids were stained with DAPI and witnessed under a confocal laser scanning microscopy (ZEISS LSM 800, German) to assess the *in vitro* tumor permeability of TR micelles and HA-TR nanosystem. In addition, C4-2B_Enz_ or RM-1_Enz_ tumoroids were treated with HA-TR@siFAM or HA@Nile-TR, respectively (Nile: 100 ng/mL, siFAM: 1 μg/mL). After 4 h of incubation, all the tumoroids were stained with DAPI and witnessed under a fluorescence microscope (Olympus IX73, Japan) to assess the *in vitro* tumor permeability of HA-TR. Moreover, C4-2B_Enz_ or RM-1_Enz_ tumoroids were treated with HA@CD36i-TR@siSCD1 and other control groups respectively, with or without 500 μM OA. After 24 h of incubation, the tumoroids witnessed under bright field with a fluorescence microscope (Olympus CKX53, Japan) to assess *in vitro* tumor ablation ability of each group.

***Quantitative real-time reverse transcription PCR (RT-qPCR)*.** Total RNA of cell or tissue samples was extracted by Trizol (Sangon Biotech, China). Subsequently, the mRNA level of CD36 or SCD1 of each group was quantified by One Step TB Green^®^ Prime Script™ PLUS RT-PCR Kit (Takara, Japan) and analyzed by QuantStudio^TM^ 6 Pro System (ThermoFisher, USA). PCR primers: human β-actin (Forward: CATGTACGTTGCTATCCAGGC, Reverse: CTCCTTAATGTCACGCACGAT), human CD36 (Forward: AACCACACACTGGGATCTGAC, Reverse: CTGCAGGAAAGTCCTACACTG), human SCD1 (Forward: TTCCTACCTGCAAGTTCTACACC, Reverse: CCGAGCTTTGTAAGAGCGGT), mouse β-actin (Forward: TTCTTGCGATACACTCTGGTGC, Reverse: CGGGATTGAATGTTCTTGTCGT), mouse CD36 (Forward: GGAACTGTGGGCTCATTGC, Reverse: CATGAGAATGCCTCCAAACAC) and mouse SCD1 (Forward: TTCTTGCGATACACTCTGGTGC, Reverse: CGGGATTGAATGTTCTTGTCGT) were purchased from Sangon Biotech, China.

***Western blotting.*** Cells or tumors were harvested in RIPA buffer (Beyotime Biotech, China) and the protein concentration was determined with a BCA kit (Beyotime Biotech, China). After dilution in loading buffer at a ratio of 4:1, lysates were boiled in a metal bath for 15 min. Then, protein samples were loaded for SDS-PAGE gel electrophoresis and transferred onto PVDF membranes (Immobilon^®^-P^SQ^, Millipore, USA). Membranes were blocked in QuickBlock™ Blocking Buffer (Beyotime Biotech, China) and probed with the anti-Tubulin beta antibody (Cat. # AF7011, Lot. # 43p2910, Affinity, 1:2000), anti-SCD1 antibody (Cat. # DF13253, Lot. # 54x0914, Affinity, 1:1000). Goat Anti-Rabbit IgG H&L (HRP) were used as secondary antibodies at the dilution 1:5000 (ab6721, 1048523-1, Abcam, UK). The blots were detected with an enhanced chemiluminescence (ECL) detection kit (Cat. # KF8005, Affinity, UK).

***In vivo* biodistribution study.** First, to develop an enzalutamide-resistant CRPC mouse model, 5-week-old C57BL/6J male mice were purchased and adapted to new circumstance for approximately one week. Then, 5 × 10^5^ RM-1_Enz_ cells were subcutaneously injected into the right hindlimb of each mouse to develop the spontaneous tumor model. After the tumors had grown to approximately 400 mm^3^, the mice were randomly divided into 3 groups (n = 3). The tumor-bearing mice were treated with free DiR, TR@DiR or HA-TR@DiR, respectively (DiR: 1 mg/kg). All mice were photographed at 6 h and 24 h by an *in vivo* animal imaging system and sacrificed after 24 h of injection. The dissected hearts, livers, spleens, lungs, kidneys and tumors were imaged with an *in vivo* animal imaging system (IVIS Spectrum, PerkinElmer) and analyzed by Living Image 4.5 software.

***In vivo* drug efficacy and safety study.** A RM-1_Enz_ cell-bearing C57BL/6J male mouse model was developed as the “*In vivo* biodistribution study” part. All the mice were randomly divided into two groups. One group was fed a low-fat diet (LFD) and one group fed a high-fat diet (HFD). When the tumor grew to ~50 mm^3^, each group of the mice were randomly divided into 6 subgroups, and treated with Saline, HA-TR, CD36i, TR@siSCD1, CD36i+TR@siSCD1 and HA@CD36i-TR@siSCD1 (CD36i: 10 mg/kg; siSCD1: 0.5 mg/kg). All mice were treated and monitored (including tumor volume and body weight) on days 14, 16, 18, 20, 22, 24 and 26, tumor volume was calculated by this equation: V = l × s^2^/2 (V: volume, l: longest diameter, s: shortest diameter). On day 26, blood samples in each group of mice were collected to determine aspartate aminotransferase (AST), alanine aminotransferase (ALT), blood urea nitrogen (BUN), and creatinine (CREA) to evaluate the safety of the drugs in each group (n = 3).

***Pathological analysis*.** After efficacy study, hearts, livers, spleens, lungs, kidneys and tumors of each group were dissected for hematoxylin-eosin staining to evaluate the toxicity of each group on major organs and the anti-tumor effect on refractory PCa. Meanwhile, tumors of each group were stained with oil red O to assess lipid accumulation in each group. Besides, in order to further evaluate the effects of drugs in each group on the proliferation/apoptosis of CRPC, Ki67/TUNEL expression levels in each group were investigated by immunofluorescence staining to evaluate the anti-proliferation effect of each group on CRPC tumors. TMR (Red) TUNEL Cell Apoptosis Detection kit, Anti-Ki67 Rabbit mAb, and HRP labeled Goat Anti-Rabbit IgG and FITC-TSA were purchased from Servicebio, China.

***Immunoactivation assay*.** To assess the remodeling effect of HA@CD36i-TR@siSCD1 on the immunomicroenvironment of refractory PCa, the tumor immunofluorescence sections of each group were dissected and prepared to evaluate the CD8^+^/CD4^+^ T cell ratio and CD4^+^FoxP3^+^ Treg cell ratio, Anti-CD8 Rabbit mAb, Anti-CD4 Rabbit mAb, Anti-FoxP3 Rabbit pAb, HRP labeled Goat Anti-Rabbit IgG, CY3-TSA and FITC-TSA were purchased from Servicebio, China. Besides, TNF-α and INF-γ levels in tumors of each group were detected with a Mouse TNF-alpha ELISA Kit (Servicebio, China) and a Mouse IFN-γ ELISA Kit (Jingmei Biotechnology, China).

***Survival analysis*.** LFD- and HFD-fed RM-1_Enz_ CRPC-bearing mouse models were established as described above and treated with Saline, HA-TR, CD36i, TR@siSCD1, CD36i+TR@siSCD1 and HA@CD36i-TR@siSCD1 (CD36i: 10 mg/kg; siSCD1: 0.5 mg/kg). All mice were treated and monitored on days 14, 16, 18, 20, 22, 24 and 26. During the survival observation period, once the tumor volume exceeded 2000 mm^3^, the mice were sacrificed as observation endpoint according to ethical principle of the Laboratory Animal Ethics Committee in Ninth People’s Hospital Affiliated to Shanghai Jiao Tong University School of Medicine.

***Targeted lipidomics study*.** The lipidomics samples was assessed by Panomix, China. Briefly, chromatographic separation was used with an ACQUITY UPLC^®^ BEH C18 (2.1 × 100 mm, 1.7 μm, Waters) column maintained at 50°C. The temperature of the autosampler was 8°C. Gradient elution of analytes was carried out with acetonitrile : water = 60:40 (0.1% formic acid + 10 mM ammonium formate) (A2) and isopropanol : acetonitrile = 90:10 (0.1% formic acid + 10 mM ammonium formate) (B2) at a flow rate of 0.25 mL/min. Injection of 2 μL of each sample was done after equilibration. Separation was conducted under the following gradient: 0~5 min, 70~57% A2; 5~5.1 min, 57%~50% A2; 5.1~14 min, 50%~30% A2; 14~14.1 min, 30% A2; 14.1~21 min, 30%~1% A2; 21~24 min, 1% A2; 24~24.1 min, 1%~70% A2; 24.1~28 min, 70% A2. The ESI-MSn experiments were used with the spray voltage of 3.5 kV and 2.5 kV in positive and negative modes, respectively. Sheath gas and auxiliary gas were set at 30 and 10 arbitrary units, respectively. The capillary temperature was 325°C. The orbitrap analyzer scanned over a mass range of m/z 150-2,000 for full scan at a mass resolution of 35,000. Data dependent acquisition (DDA) MS/MS experiments were performed with HCD scan. The normalized collision energy was 30 eV. Dynamic exclusion was implemented to remove some unnecessary information in MS/MS spectra.

***Statistical analysis*.** Statistical analyses were performed using GraphPad Prism 8.2.1. All values are expressed as the mean ± SD. Comparisons between multiple groups were analyzed by one- or two-way ANOVA. A *p* value < 0.05 was considered statistically significant.

**Figure S1.** The synthetic route of TR peptide.

**Figure S2.** HPLC chromatogram of TR peptide.

**Figure S3.** Mass spectrum of TR peptide.

**Figure S4.** Peptide rkkrrqrrrHlKYDGR sequencing results. **(A)** Total ion flow chromatogram of rkkrrqrrrHlKYDGR peptide; **(B)** Primary mass spectrum map of rkkrrqrrrHlKYDGR peptide; **(C)** Mass spectrum results of rkkrrqrrrHlKYDGR peptide calculated based on primary mass spectrometry; **(D)** Second-order mass spectrum map of rkkrrqrrrHlKYDGR peptide; **(E)** Matching fragment ion peak table (matching fragment ion peak marked with red).

**Figure S5.** Construction of two drug-resistant cell lines PC-3_Enz_ and RM-1_Enz_. **(A)** Cell cloning results of RM-1 and PC-3 cell lines sensitive to enzalutamide and PC-3_Enz_ and RM-1_Enz_-resistant cell lines constructed under normal culture conditions or in the presence of 40 μM enzalutamide (500 cells/well, cultured for 2 weeks); **(B, C)** Cell survival curves and IC_50_ values of RM-1/RM-1_Enz_ and PC-3/PC-3_Enz_ under 0-200 μg/mL concentration gradients (n = 3, mean ± SD).

**Figure S6.** Toxicity of the HA-TR delivery system on PC-3_Enz_, RM-1_Enz_, C4-2B_Enz_ cells (n = 3, mean ± SD).

**Figure S7.** *In vitro* tumor penetration study. (A, B) Study on tumoroid penetration ability of HA-TR@siFAM in C4-2B_Enz_ or RM-1_Enz_ tumoroids (bars = 500 μm); (C, D) Study on tumoroid penetration ability of HA@Nile-TR in C4-2B_Enz_ or RM-1_Enz_ tumoroids (bars = 50 μm).

**Figure S8.** The expression rates of CD36 on the cell membrane of C4-2B_Enz_ or RM-1_Enz_ cells, comparing with C4-2B or RM-1 cells (n = 3, one-way ANOVA, *****p* < 0.0001).

**Figure S9.** Dose-effect plots and combination index (CI) – faction effect (Fa) plots generated by CompuSyn software to calculate the synergistic effect activity combining CD36i with siSCD1 in CD36i + TR@siSCD1 group or HA@CD36i-TR@siSCD1 group. Administration groups were co-incubated with C4-2B_Enz_ or RM-1_Enz_ cells for 24 h, with or without 500 μM OA.

**Figure S10.** Anti-proliferation test of C4-2B_Enz_ and RM-1_Enz_ tumoroids (CD36i: 20 μg/mL, siSCD1: 100 nM, bars = 500 μm).

**Figure S11.** Anti-proliferation test of C4-2B_Enz_ and RM-1_Enz_ tumoroids, co-incubated with 500 μM OA (CD36i: 20 μg/mL, siSCD1: 100 nM, bars = 500 μm).

**Figure S12.** Cellular apoptosis analysis. **(A)** C4-2B_Enz_ and **(B)** RM-1_Enz_ cells were incubated with HA@CD36i-TR@siSCD1 and other control groups for 24 h (CD36i: 20 μg/mL, siSCD1: 100 nM, bars = 20 μm).

**Figure S13.** *In vitro* analysis of cell oil red O staining (CD36i: 20 μg/mL, siSCD1: 100 nM, bars = 20 μm).

**Figure S14.** RT-qPCR analysis of CD36 and SCD1 mRNA levels in RM-1_Enz_ cells (n = 3, mean ± SD, one-way ANOVA, n.s.: no significance, ** *p* < 0.01, *** *p* < 0.001).

**Figure S15.** Western blotting analysis of SCD1 expression in **(A)** LFD-fed mice and **(B)** HFD-fed mice (n = 3, mean ± SD, one-way ANOVA, * *p* < 0.05, ** *p* < 0.01, *** *p* < 0.001).

**Figure S16.** Basic and multivariate statistical analysis of lipidomics. **(A)** Global lipid correlation clustering heatmap; **(B-J)** Multivariate statistical analysis of lipidomics (LFD model, group B *v.s*. group A, group A: PBS group, group B: HA@CD36i-TR@siSCD1 group, n = 4); **(B)** OPLS-DA S-plot; **(C)** OPLS-DA score plot; **(D)** OPLS-DA loading plot; **(E)** OPLS-DA permutation test diagram; **(F)** PCA score plot; **(G)** PCA loading plot; **(H)** PLS-DA plot; **(I)** PLS-DA permutation test diagram; **(J)** PLS-Da loading plot; **(K-S)** Multivariate statistical analysis of lipidomics (HFD model, group D *v.s*. group C, group C: PBS group, group D: HA@CD36i-TR@siSCD1 group, n = 4); **(K)** OPLS-DA S-plot; **(L)** OPLS-DA score plot; **(M)** OPLS-DA loading plot; **(N)** OPLS-DA permutation test diagram; **(O)** PCA score plot; **(P)** PCA loading plot; **(Q)** PLS-DA plot; **(R)** PLS-DA permutation test diagram; **(S)** PLS-Da loading plot.

**Figure S17.** Typical HE-staining images of heart, liver, spleen, lung, kidney and tumor of each group (400×).

| **Group** | **IC_50_** | |
| --- | --- | --- |
|  | CD36i (μg*mL^-1^) | siSCD1 (nM) |
| **CD36i** | 18.36 | - |
| **TR@siSCD1** | - | 67.47 |
| **CD36i+TR@siSCD1** | 4.267 | 42.67 |
| **HA@CD36i-TR@siSCD1** | 2.915 | 29.15 |

**Table S1.** IC_50_ values corresponding to CD36i and siSCD1 in each administration group of C4-2B_Enz_.

| **Group** | **IC_50_** | |
| --- | --- | --- |
|  | CD36i (μg*mL^-1^) | siSCD1 (nM) |
| **CD36i** | 17.70 | - |
| **TR@siSCD1** | - | 855.2 |
| **CD36i+TR@siSCD1** | 7.386 | 73.86 |
| **HA@CD36i-TR@siSCD1** | 3.038 | 30.38 |

**Table S2.** IC_50_ values corresponding to CD36i and siSCD1 in each administration group of C4-2B_Enz_ + OA (OA: 500 μM).

| **Group** | **IC_50_** | |
| --- | --- | --- |
|  | CD36i (μg*mL^-1^) | siSCD1 (nM) |
| **CD36i** | 9.935 | - |
| **TR@siSCD1** | - | 91.30 |
| **CD36i+TR@siSCD1** | 3.478 | 34.78 |
| **HA@CD36i-TR@siSCD1** | 2.701 | 27.01 |

**Table S3.** IC_50_ values corresponding to CD36i and siSCD1 in each administration group of RM-1_Enz_.

| **Group** | **IC_50_** | |
| --- | --- | --- |
|  | CD36i (μg*mL^-1^) | siSCD1 (nM) |
| **CD36i** | 21.26 | - |
| **TR@siSCD1** | - | 746.6 |
| **CD36i+TR@siSCD1** | 6.784 | 67.84 |
| **HA@CD36i-TR@siSCD1** | 4.081 | 40.81 |

**Table S4.** IC_50_ values corresponding to CD36i and siSCD1 in each administration group of RM-1_Enz_ + OA (OA: 500 μM).

| **Group** | **CI value** | |
| --- | --- | --- |
|  | CD36i+TR@siSCD1 | HA@CD36i-TR@siSCD1 |
| **C4-2B_Enz_** | 0.83^△^ | 0.64^△△^ |
| **C4-2B_Enz_ + OA** | 0.69^△△^ | 0.19^△△△^ |
| **RM-1_Enz_** | 0.81^△^ | 0.72^△^ |
| **RM-1_Enz_ + OA** | 0.64^△△^ | 0.39^△△^ |

**Table S5.** CI values (Fa = 0.5) corresponding to CD36i+TR@siSCD1 and HA@CD36i-TR@siSCD1 for C4-2B_Enz_ or RM-1_Enz_ cells (with or without 500 μM OA, ^△^ 0.7 < CI ≤ 0.85: Moderate Synergism, ^△△^ 0.3< CI ≤ 0.7: Synergism, ^△△△^ 0.1 < CI ≤ 0.3: Strong Synergism).

| **Group** | **Median Survival (d)** | |
| --- | --- | --- |
|  | LFD | HFD |
| **PBS** | 23 | 21 |
| **HA-TR** | 25 | 21 |
| **CD36i** | 26 | 33 |
| **TR@siSCD1** | 30 | 36 |
| **CD36i+TR@siSCD1** | 39 | 42 |
| **HA@CD36i-TR@siSCD1** | 42 | 54 |

**Table S6.** Median survival of each group of LFD-fed or HFD-fed mice.

**References**

[1] Y. Wang, J. Chen, L. Gong, Y. Wang, A. Siltari, Y. R. Lou, T. J. Murtola, S. Gao, Y. Gao. *J. Nanobiotechnology* **2024**, 22(1), 145.

[2] J. Chen, Y. Wang, L. Han, R. Wang, C. Gong, G. Yang, Z. Li, S. Gao, Y. Yuan. *Mater. Today Bio* **2022**, 17, 100484.
